# Stemness- and hypoxia-based prognostic stratification index reveals G6PD as a regulator of hypoxia-driven stemness in hepatocellular carcinoma

**DOI:** 10.3389/fimmu.2025.1669275

**Published:** 2025-09-19

**Authors:** Mingwei Gao, Yuechuan Liu, Jianhui Wu, Peiru Zhang, Jin Liu, Kun Guo, Binwen Sun, Sunbin Ling, Liming Wang

**Affiliations:** ^1^ Division of Hepatobiliary and Pancreatic Surgery, Department of General Surgery, The Second Affiliated Hospital of Dalian Medical University, Dalian, China; ^2^ Engineering Research Center for New Materials and Precision Treatment Technology of Malignant Tumors Therapy, The Second Affiliated Hospital of Dalian Medical University, Dalian, China; ^3^ Engineering Technology Research Center for Translational Medicine, The Second Affiliated Hospital of Dalian Medical University, Dalian, China

**Keywords:** hepatocellular carcinoma, stemness, hypoxia, prognosis, immune microenvironment, G6PD

## Abstract

**Background:**

The positive feedback loop between cancer stemness and the hypoxic microenvironment is a critical driver of hepatocellular carcinoma (HCC) progression. Analyzing their interaction in HCC is crucial to characterize immune microenvironment features, uncover molecular heterogeneity patterns, and develop targeted interventions.

**Methods:**

The TCGA-LIHC cohort (n=340) were stratified through consensus clustering of stemness- and hypoxia-related genes (SHRGs) identified by one-class logistic regression and weighted gene co-expression network analyses. Subsequently, a stemness- and hypoxia-related prognostic index (SHRPI) was constructed using random forest, and Cox regression analyses, with its prognostic significance assessed in two other independent cohorts: our NC-LT cohort comprising 180 liver transplant (LT) patients with HCC beyond Milan criteria, and the GSE104580 cohort containing 147 HCC patients treated with transcatheter arterial chemoembolization (TACE). A prognostic nomogram incorporating SHRPI was developed, and externally validated in the GSE14520 cohort (n=242). Systematic profiling of immune microenvironment features and immunotherapy responsiveness in SHRPI subgroups was performed, followed by pharmacogenomic screening and molecular docking to identify optimal therapies. After single-cell transcriptomic analysis, functional validation assays were conducted to confirm the role of G6PD, a key SHRPI component.

**Results:**

SHRGs-based clustering revealed two clusters exhibiting distinct prognoses, functional annotations, genomic alterations, and immune microenvironment features. SHRPI served as an independent risk factor for both overall survival in HCC patients and recurrence-free survival in LT patients beyond Milan criteria. It demonstrated strong predictive power for TACE responsiveness. The SHRPI-integrated nomogram achieved robust performance in external validation. High SHRPI level was associated with a more immunosuppressive tumor microenvironment and poorer immunotherapy responsiveness. Pharmacogenomic and molecular docking analyses identified BI2536 as the most promising therapeutic agent for this high-SHRPI subgroup. Further experiments established that G6PD serves as a key therapeutic target for hypoxia-driven stemness maintenance in HCC by functioning as a stemness regulator that interacts with HIF-1α to form a positive feedback loop under hypoxia.

**Conclusions:**

This study provides further insights into stemness-hypoxia interaction in HCC and delivers a clinically applicable predictive tool for prognosis. BI2536’s synergy potential and the therapeutic value of G6PD targeting in stemness regulation advance individualized therapeutic strategies for HCC.

## 1 Introduction

Hepatocellular carcinoma (HCC) is a highly aggressive malignancy characterized by a high recurrence rate and broad therapeutic resistance, significantly impacting patient prognosis (1). Studies have shown that the malignant characteristics of HCC are closely linked to the presence and function of cancer stem cells (CSCs) (2, 3). CSCs possess self-renewal capacity and differentiation plasticity, enabling them to evade immune surveillance and play a pivotal role in tumor initiation, progression, and therapeutic resistance (4, 5). These cells maintain their stem-like properties by activating embryonic developmental signaling pathways such as Wnt/β-catenin and Notch while upregulating drug efflux pumps, thereby enhancing resistance to conventional therapies (6, 7). As reservoirs for tumor relapse, CSCs rely heavily on the hypoxic tumor microenvironment (TME) for survival and stemness maintenance (8, 9).

Within solid tumors, low oxygen levels not only promote angiogenesis and metabolic reprogramming to support tumor growth but also activate hypoxia-inducible factors (HIFs), which transcriptionally upregulate stemness-related genes such as Oct4 and Nanog. This process further sustains the stem-like phenotype of CSCs, contributing to HCC invasiveness and treatment resistance (10–13). Moreover, hypoxia reprograms the TME into an immunosuppressive niche, amplifying CSC-driven malignancy. Intratumoral hypoxia induces the secretion of cytokines such as TGF-β and IL-6 while promoting the recruitment of regulatory T cells (Tregs) and myeloid-derived suppressor cells (MDSCs). These immunosuppressive components cooperatively inhibit cytotoxic T-cell (CD8^+^ T-cell) activity, protecting CSCs from immune clearance (14). Additionally, hypoxia-driven upregulation of immune checkpoints such as PD-L1 and CTLA-4 exacerbates immune evasion (15, 16). As the tumor progresses, increased oxygen consumption exacerbates TME hypoxia, forming a self-reinforcing malignant cycle that enhances tumor aggressiveness (8, 17). Therefore, identifying, quantifying, and therapeutically targeting stemness-hypoxia features holds significant clinical value for optimizing HCC risk stratification and precision therapy.

Despite the recognized role of the stemness-hypoxia axis in HCC progression, there remains a lack of systematic, rigorous, and effective prognostic indices that integrate stemness and hypoxia characteristics for stratifying patients and identifying high-risk subgroups to guide precision treatment strategies. Furthermore, current HCC prognostic models are primarily based on either single molecular features (e.g., stemness indices or hypoxia scores) or clinicopathological parameters (18–21). The limited dimensionality of these models restricts their accuracy in predicting patient outcomes. Therefore, it is imperative to develop a comprehensive prognostic tool that integrates multi-dimensional molecular features (incorporating both stemness and hypoxia) with clinicopathological parameters to improve risk stratification, enhance prediction accuracy, and provide a rationale for personalized treatment strategies for high-risk subgroups.

In this study, we utilized large-scale public multi-omics datasets and integrated multiple machine learning algorithms and statistical analysis methods to identify key genes involved in stemness-hypoxia regulation. We constructed a stemness- and hypoxia-related prognostic index (SHRPI) to stratify HCC patients into distinct risk subgroups, and developed a high-performance prognostic nomogram. Additionally, we screened potential therapeutic drugs targeting high-risk subgroup through pharmacogenomic and molecular docking analyses. Capitalizing on the high-resolution cellular heterogeneity mapping capability of single-cell transcriptomics (22, 23), we further dissected the cell-type-specific expression profiles of SHRPI components and validated the role and potential mechanism of its most critical gene in maintaining HCC stemness under hypoxia.

## 2 Materials and methods

### 2.1 Data collection and preprocessing

The discovery cohort of HCC patients was obtained from TCGA-LIHC through their data portal (https://portal.gdc.cancer.gov/projects/TCGA-LIHC), comprising gene expression profiles, copy number variation (CNV) data, somatic mutation data, and clinical information. The validation cohort consisted of gene expression profiles and clinical information from the GSE14520 dataset, retrieved from GEO database (https://www.ncbi.nlm.nih.gov/geo/). After comprehensive screening, this study included 340 patients from TCGA-LIHC with complete survival information, overall survival (OS) > 30 days, and accessible stemness indices and hypoxia scores, as well as 242 patients from GSE14520 fulfilling the criteria of complete survival data and OS > 30 days. For transcriptomic data normalization, log2(FPKM + 0.001) transformation was applied. To mitigate batch effects in transcriptomic data, we followed the recommended standard procedures for bulk transcriptomic data analysis in cancer research, applying the Combat algorithm from the “SVA” R package for batch effect correction (23). Additionally, we incorporated 180 liver transplant (LT) patients with HCC beyond the Milan criteria from our previous study (NC-LT cohort) to evaluate the impact of the gene signature on recurrence-free survival (RFS) (24), along with 147 patients from the GSE104580 dataset to assess the correlation between the gene signature and patient response to transcatheter arterial chemoembolization (TACE) therapy.

### 2.2 Computation of stemness indices

The stemness signature was determined using the one-class logistic regression (OCLR) machine-learning algorithm (25). Subsequently, correlation coefficients were computed between the stemness signature weight values and gene expression levels for each sample. Finally, the stemness index was derived by scaling the Spearman correlation coefficients to a range between 0 and 1.

### 2.3 Differential expression analysis

The TCGA-LIHC samples were categorized into high and low groups based on either the median value or the optimal cutoff value determined by maximizing the Youden index using the “survminer” R package. Differential expression analysis was conducted using the Wilcoxon rank-sum test (26). Genes meeting the criteria of false discovery rate (FDR) < 0.05 and |log2(fold change)| > 1 were considered statistically significant. To enhance the accuracy of the risk model, a more stringent selection threshold was applied, setting FDR < 0.01 and |log2(fold change)| > 2.

### 2.4 Definition of stemness- and hypoxia-related genes

The hypoxia signature score for TCGA-LIHC patients was obtained from The cBio Cancer Genomics Portal (http://cbioportal.org), and hypoxia-related genes were identified using weighted gene co-expression network analysis (WGCNA) (27). The overlapping genes between mRNAsi-related differentially expressed genes (DEGs) and hypoxia-related genes were collectively defined as stemness- and hypoxia-related genes (SHRGs).

### 2.5 Unsupervised consensus clustering

The “ConsensusClusterPlus” R package was employed for the classification of SHRGs through unsupervised consensus clustering. To enhance classification stability, the clustering process was conducted 1,000 times with 80% resampling. The optimal k value (number of clusters) was identified based on the relative variation in the area under the cumulative distribution function (CDF) curves and the consensus matrix.

### 2.6 Functional enrichment analysis

GO, KEGG, and GSEA analyses were conducted using the “clusterProfiler” R package, while GSVA analysis was performed with the unsupervised “GSVA” R package. The background gene sets for both GSEA and GSVA were obtained from the Molecular Signatures Database (MSigDB) (28), specifically the h.all.v2024.1.Hs.symbols.gmt gene set. Subsequently, differential analysis of the GSVA results was conducted using the “limma” R package, considering pathways with FDR < 0.05 as significantly enriched, with |t| > 2 shown in figures for visualization.

### 2.7 Genetic alterations and immune infiltration analysis

The CNV and somatic mutation data of TCGA-LIHC patients were analyzed using the “maftools” R package to examine genetic alterations across different clusters. The 14 oncogenic pathways were compared across various clusters using the PROGENy algorithm (29). To evaluate the tumor immune microenvironment (TIME), the “Cell-type Identification by Estimating Relative Subsets of RNA Transcripts (CIBERSORT)” tool was employed to quantify the abundance of tumor-infiltrating immune cells (30).

### 2.8 Immune checkpoints and immunotherapy response analysis

To assess immunotherapy response in TCGA-LIHC patients, expression of 68 immune checkpoint-related genes identified in previous studies was analyzed (31). Subsequently, tumor immune dysfunction and exclusion (TIDE) scores, T cell dysfunction scores, T cell exclusion scores, INFG levels, and MDSC levels were retrieved from the TIDE portal (http://tide.dfci.harvard.edu). Single-sample gene set enrichment analysis (ssGSEA) was then applied to compute enrichment scores for 29 immune-related traits and to explore associations between the index and immune regulation (32). Furthermore, ssGSEA-derived enrichment scores for three stem cell related gene sets from MSigDB: “WONG EMBRYONIC STEM CELL CORE,” “YAMASHITA LIVER CANCER STEM CELL UP,” and “YAMASHITA LIVER CANCER STEM CELL DN” were calculated to investigate associations between the index and stemness.

### 2.9 Construction of SHRPI

To determine the relationship between SHRGs and patient survival outcomes, we applied univariate Cox regression, LASSO regression, and Random Forest models to filter SHRGs. After excluding attributes with an absolute correlation of 0.8, a total of 419 genes were selected as input variables. Finally, the four most critical genes were identified and incorporated into a multivariate Cox regression model to construct a risk prediction model, termed SHRPI. The formula for this model is as follows:


$$\text{Risk score}=\sum_{i=0}^{n}(\text{Coefficient}(\text{i})\times \text{ Expression }(\text{i}))$$

To evaluate the robustness of SHRPI, patients were initially stratified into two groups based on the median SHRPI value. The prognostic significance of SHRPI was assessed using Kaplan-Meier survival analysis. The predictive accuracy of SHRPI was further evaluated through receiver operating characteristic (ROC) curve analysis, with the area under the curve (AUC) calculated using the “timeROC” R package. To enhance its clinical applicability, TCGA-LIHC patients were further categorized into high-risk (HRG) and low-risk (LRG) groups based on the optimal cutoff value, followed by comprehensive immune profiling and drug sensitivity analyses.

### 2.10 Construction of nomogram predictive model

The SHRPI score, along with tumor stage, age, gender, tumor grade, vascular invasion status, Child-Pugh grade, hepatic inflammation status, cirrhosis status, recurrence status, BMI, and AFP levels, was incorporated into the univariate Cox regression analysis. The hazard ratios (HRs) for each variable were computed using the Cox proportional hazards regression model with the “survival” R package. To determine independent prognostic factors, a multivariate Cox regression analysis was performed, and a nomogram was developed based on the findings using the “RMS” R package. Model stability was assessed through Schoenfeld residuals and deviance residuals. The nomogram’s predictive performance was evaluated via ROC analysis, calibration curves, and the C-index, calculated through 1,000 bootstrap resampling iterations. Furthermore, decision curve analysis (DCA) was employed to assess the clinical applicability of the predictive model (33).

### 2.11 Drug response analysis and molecular docking analysis

Gene expression data, along with the corresponding half-maximal inhibitory concentration (IC50) values and area under the dose-response curve (AUC) for cancer cell lines, were obtained from the Genomics of Drug Sensitivity in Cancer (GDSC2 v8.5, released October 2023), the Cancer Therapeutics Response Portal (CTRP v2.0, released October 2015), and the Profiling Relative Inhibition Simultaneously in Mixtures (PRISM) Repurposing dataset (20Q2, released August 2022). AUC values were used as a measure of drug sensitivity, where higher AUC values indicated lower treatment sensitivity. The “oncoPredict” R package was employed to predict drug sensitivity for each sample.

The molecular structures of the compounds were retrieved from PubChem Compound (https://pubchem.ncbi.nlm.nih.gov/), and the 3D coordinates of G6PD (PDB ID: 7UAG, resolution: 3.5Å) were obtained from the PDB (http://www.rcsb.org/). All protein and molecular files were converted into PDBQT format, with water molecules removed and polar hydrogen atoms added to improve docking accuracy. Molecular docking simulations were conducted using AutoDock Vina 1.2.2, and the resulting protein–ligand complexes were visualized with PyMol. The binding energies, which indicate binding stability, were used to assess the therapeutic potential of each compound (34).

### 2.12 Single‐cell RNA sequencing analysis

Single-cell RNA sequencing data from HCC samples in GEO dataset GSE149614 were filtered to remove low-quality cells (< 200 or > 6,000 detected genes, or > 15% mitochondrial content). Gene expression was log-normalized using Seurat (v5.3.0), followed by PCA for dimensionality reduction and clustering via the FindNeighbors and FindClusters functions. Cell clusters were annotated using the “SingleR” R package. SHRPI was computed as a weighted sum of z-score-normalized expression of HMMR, UBE2S, G6PD, and NEIL3. Cells were classified into low- and high-SHRPI groups based on the median SHRPI score. SHRPI distribution was visualized on the t-SNE plot, and expression of each constituent gene across cell clusters was displayed in separate dot plots.

### 2.13 Cell culture, lentiviral vector construction and infection

Human HCC cell lines (HuH-7, PLC/PRF/5, Hep-3B, and Li-7) and HEK293 were obtained from the Shanghai Institute of Cell Biology, Chinese Academy of Sciences (Shanghai, China). The authenticity of all cells’ authenticity was confirmed through short-tandem repeat (STR) profiling. The cells were cultured in the appropriate media supplemented with 10% fetal bovine serum and 1% penicillin-streptomycin (Solarbio) at 37 °C in a 5% CO_2_ incubator. Hep-3B and PLC/PRF/5 cells were maintained in MEM medium (Pricella), HEK293 and HuH-7 cells were cultured in high-glucose DMEM (Pricella), and Li-7 cells were maintained in RPMI 1640 medium (Pricella). All cell lines were regularly confirmed to be mycoplasma-free by PCR. Detailed information on lentiviral vector construction and infection is provided in Supplementary File, and all plasmid sequences are listed in **Supplementary Table 3**.

### 2.14 Cell migration assay

Cells (2×10^4^) were seeded in the upper chamber of a polycarbonate membrane insert (Corning Incorporated) with 200 μL of FBS-free medium. The lower chamber was filled with 800 μL of medium containing 20% FBS. After 24–48 hours of incubation, the cells that had migrated through the membrane were washed, fixed with 1% paraformaldehyde, and stained with crystal violet. Photographs were taken of four randomly selected fields, and the number of migrated cells was counted. The experiment was performed in triplicate.

### 2.15 Cell sphere formation assay

Cells (2×10^3^) were plated onto 6-well Ultra-Low Attachment plates (Corning Incorporated) and cultured in special medium consisting of DMEM/F12 (Invitrogen) supplemented with 4 μg/mL insulin (Sigma-Aldrich), B27 (Invitrogen), 20 ng/mL EGF (Sigma-Aldrich), and 20 ng/mL basic FGF (Invitrogen). After 10 days of incubation, spheres with diameters greater than 75 μm were photographed and counted under a microscope.

### 2.16 Western blot analysis, co-immunoprecipitation and quantitative PCR analysis

Detailed information is provided in **Supplementary File**. Specific details regarding the antibodies are presented in **Supplementary Table 4**. The sequences of primers are provided in **Supplementary Table 5**.

### 2.17 Hydrodynamic tail vein injection mouse model

The transgenic HCC mouse model was generated in male wild-type C57BL/6J mice (6–8 weeks) by hydrodynamic tail vein injection co-overexpressing activated AKT and c-Met. In brief, the plasmids m-G6PD pT3-EF1α-MYC or pT3-EF1α-MYC (MCS) (20 μg), pT3-myr-AKT-HA (20 μg), and pT3-EF1α-c-Met (20 μg), together with pCMV(CAT)T7-SB100 (2.4 μg), at a ratio of 12.5: 12.5: 12.5: 1.5, were diluted in 2 ml saline (0.9% NaCl), filtered through a 0.22-μm filter, and injected into the lateral tail vein of the mice within 5-7s (35). 20 days after injection, the mice were humanely euthanized via intraperitoneal injection of sodium pentobarbital (150 mg/kg). Liver tumors were subsequently collected for analysis. Animal experiments were conducted in strict accordance with relevant guidelines and approved by the Institutional Animal Care and Use Committee of Zhejiang Center of Laboratory Animals (IACUC, ZJCLA; approval number: ZJCLA-IACUC-20011186). This study adhered to the ARRIVE guidelines.

### 2.18 Statistical analysis

The Student’s t-test or Wilcoxon rank-sum test (Mann-Whitney U test) was applied to assess continuously distributed numerical data. Correlation analysis was conducted using either the Pearson or Spearman correlation test, depending on data distribution. Survival curves were generated with the Kaplan-Meier method and compared using the Log-rank test. All statistical analyses were performed in GraphPad Prism (v9.0) and R (v4.4.1). A two-tailed *P* value < 0.05 was considered statistically significant. Statistical significance was annotated as follows: *P < 0.05, **P < 0.01, ***P < 0.001, ****P < 0.0001; ns = not significant.

## 3 Results

### 3.1 Stemness indices and hypoxia scores in HCC

Using mRNA expression and DNA methylation data from the TCGA-LIHC cohort, five stemness indices were calculated, with hypoxia scores obtained from the cBioPortal database. The mRNA expression-based stemness index (mRNAsi) (*P* = 0.0031) and the Buffa Hypoxia Score (*P* < 0.0001) showed significant associations with OS in HCC (**Supplementary Figures 1A, D**). Patients with higher mRNAsi exhibited significantly poorer tumor differentiation (*P* < 0.0001), increased vascular invasion (*P* = 0.022), and elevated AFP levels (*P* = 0.021) (**Supplementary Figures 1B, C**). Similarly, a higher Buffa Hypoxia Score was associated with advanced tumor stage (*P* < 0.0001), poorer tumor differentiation (*P* = 0.006), increased vascular invasion (*P* = 0.007), and elevated AFP levels (*P* = 0.027) (**Supplementary Figures 1E, F**). Thus, mRNAsi was selected to quantify stemness characteristics, while the Buffa Hypoxia Score was used to evaluate tumor hypoxia levels.

### 3.2 Identification of stemness- and hypoxia-related clusters in HCC

**Figure 1:**
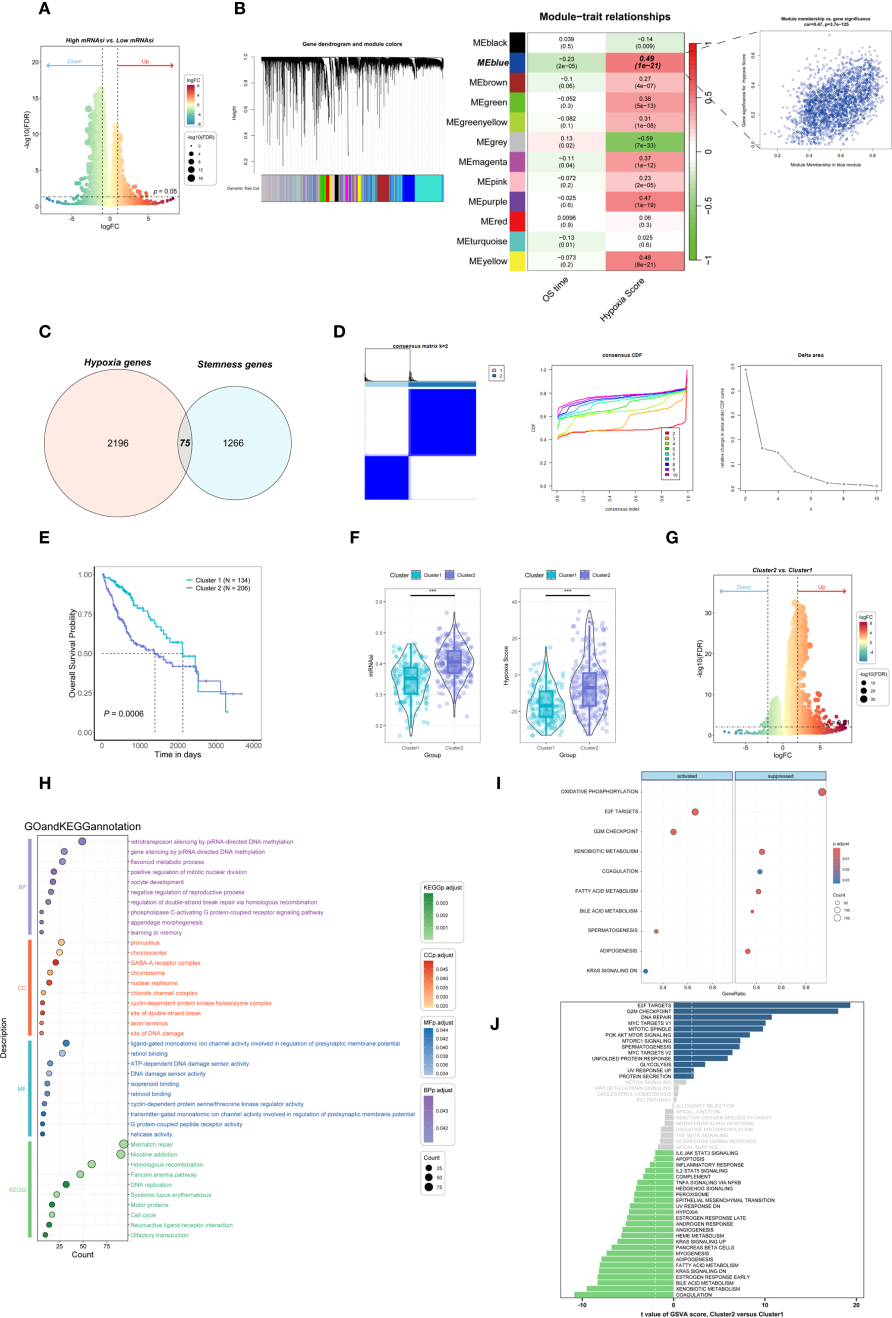
Identification of stemness- and hypoxia-related clusters. **(A)** Identification of mRNAsi-related DEGs between high- and low-mRNAsi subgroups. **(B)** Hypoxia-related genes identified through WGCNA. **(C)** A total of 75 overlapping genes were identified as SHRGs. **(D)** Unsupervised consensus clustering was performed using SHRGs. **(E)** Kaplan-Meier survival curve for the identified clusters. **(F)** Differences in mRNAsi (left) and hypoxia scores (right) between the two clusters. **(G)** Identification of DEGs between the two clusters. **(H–J)** KEGG/GO **(H)**, GSEA **(I)**, and GSVA **(J)** enrichment analyses of the two clusters. Statistical significance: ***P < 0.001.

HCC patients were categorized into high- and low-stemness groups based on the median mRNAsi value, resulting in the identification of 1,341 DEGs associated with mRNAsi (**Figure 1A**). WGCNA revealed 11 non-grey modules, with the blue module showing the strongest correlation with the Buffa Hypoxia Score (R² = 0.47, *P* = 3.7×10^−125^) (**Figure 1B**). Integrating mRNAsi-associated DEGs with hypoxia-related genes identified 75 overlapping genes, classified as SHRGs (**Figure 1C**, **Supplementary Table 1**). Consensus clustering was performed on the 75 SHRGs, determining optimal classification at k = 2, as indicated by the CDF curve variations (**Figure 1D**). HCC patients were then divided into two clusters (Cluster 1 and Cluster 2). Patients in Cluster 2 exhibited a significantly shorter median OS and lower survival probability than those in Cluster 1 (*P* = 0.0006) (**Figure 1E**), as well as higher mRNAsi and hypoxia scores (both *P* < 0.001) (**Figure 1F**).

To further characterize the molecular differences between the two clusters, we conducted a more stringent differential expression analysis using |log2FC| > 2 and FDR < 0.01 as the selection criteria (**Figure 1G**, **Supplementary Figure 2A**). Functional enrichment analysis of DEGs through GO and KEGG revealed significant enrichment in cell cycle regulation and DNA repair pathways (**Figure 1H**). GSEA further demonstrated that, compared with Cluster 1, Cluster 2 exhibited significant activation of E2F targets, the G2/M checkpoint, and KRAS signaling DN, whereas oxidative phosphorylation, bile acid metabolism, fatty acid metabolism, and adipogenesis were suppressed (**Figure 1I**). GSVA revealed significant upregulation of proliferation-related pathways, including E2F targets, G2/M checkpoint, DNA repair, and mTOR signaling in Cluster 2. In contrast, Cluster 1 was predominantly enriched in lipid and bile acid metabolism, coagulation, and inflammatory responses (**Figure 1J**). Overall, these results indicate that Cluster 2 is characterized by a hyperproliferative phenotype with enhanced DNA repair and oncogenic signaling, whereas Cluster 1 is associated with metabolic reprogramming and an inflammatory tumor microenvironment.

### 3.3 Genomic and TIME characteristics of the two stemness- and hypoxia-related clusters

**Figure 2:**
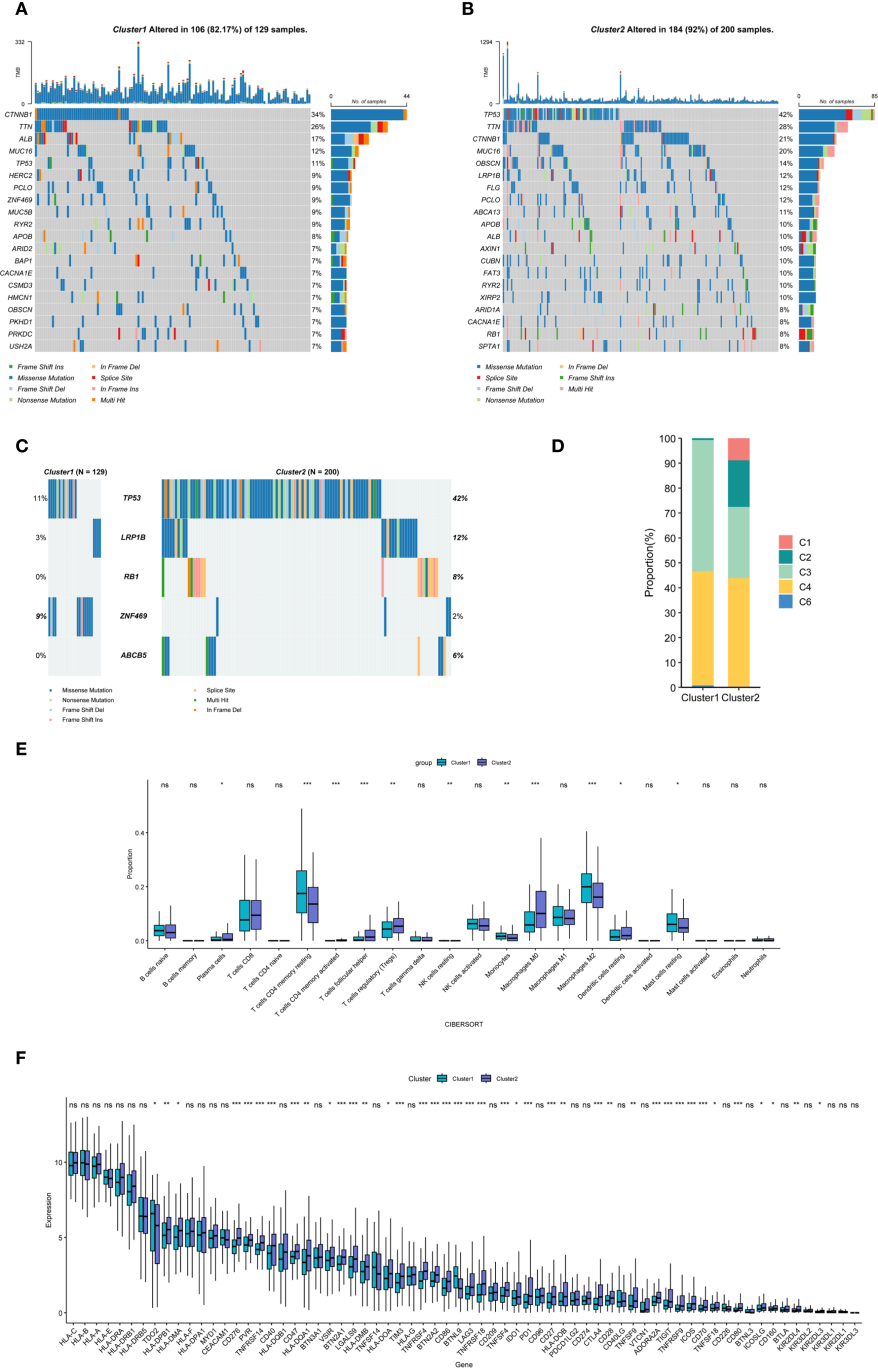
Genomic and TIME characteristics of the two stemness- and hypoxia-related clusters. **(A, B)** Waterfall plots illustrating the twenty most frequently mutated genes in each cluster. **(C)** Mutation landscape of the five most significantly different genes based on univariate Cox analysis in the two clusters. **(D)** Differences in the composition of previously classified molecular subtypes of HCC between the two clusters. **(E)** Comparison of immune cell infiltration proportions between the two clusters. **(F)** Analysis of expression levels of representative immune checkpoint genes across the two clusters. Statistical significance: *P < 0.05, **P < 0.01, ***P < 0.001; ns = not significant.

We next examined somatic mutations and CNV in both clusters to investigate potential mechanisms underlying their distinct prognoses. Recurrent mutations were detected in several genes, including TP53, TTN, and CTNNB1, and the two clusters exhibited distinct single-nucleotide variant (SNV) substitution patterns (**Figures 2A, B**, **Supplementary Figures 2E, F**). Notably, TP53, LRP1B, RB1, ABCB5, and ZNF469 displayed significantly different mutation frequencies between the clusters (**Figure 2C**, **Supplementary Figures 2B, C**). Moreover, Cluster 2 exhibited higher aneuploidy scores, tumor mutational burden (TMB) and homologous recombination deficiency (HRD) compared with Cluster 1 (**Supplementary Figure 2D**). These findings suggest that tumors with elevated DNA damage may possess enhanced immune evasion capabilities and reduced responsiveness to immunotherapy (36). To further validate our classification, we compared our patient clusters with a previously established molecular classification in which the “Inflammatory” subtype (C3) was associated with the best prognosis (37). Most C3 patients were assigned to Cluster 1, which had a better prognosis, consistent with previous findings (**Figure 2D**).

Given the intricate interplay between stemness, hypoxia, and immune-related pathways, we further explored differences in the TIME across the two clusters. CIBERSORT analysis revealed that patients in Cluster 2 exhibited significantly higher levels of activated memory CD4^+^ T cells, follicular helper T cells, and Tregs, whereas M0 macrophages, resting memory CD4^+^ T cells, and M2 macrophages were markedly reduced (**Figure 2E**). Next, we analyzed the expression of previously reported immune checkpoint-related genes across the two clusters (38). In Cluster 2, genes known to suppress T-cell immune activity, including CTLA4 and its ligands as well as PD-1 and its ligands, were significantly upregulated (**Figure 2F**). With the growing prominence of immunotherapy in HCC treatment, we employed the TIDE model to evaluate patients’ potential response. Given that higher TIDE scores indicate increased immune evasion and diminished immunotherapy efficacy, we observed that Cluster 2 had a significantly higher TIDE score than Cluster 1, suggesting that patients in Cluster 2 may have a lower likelihood of benefiting from immunotherapy (**Supplementary Figure 3A**). We employed ssGSEA to evaluate therapeutic signatures and 29 immune-related gene signatures, encompassing immune, stromal, and other cellular processes. First, Cluster 2 exhibited significant upregulation of gene signatures associated with the cell cycle, DNA replication, and mismatch repair (**Supplementary Figure 3B**). Second, Cluster 2 displayed increased infiltration of immunosuppressive cells, including tumor-associated macrophages (TAMs), MDSCs and Tregs, along with enhanced tumor cell proliferation, leading to heightened immune suppression and elevated pro-tumor immune scores (**Supplementary Figure 3E**). Finally, PROGENy analysis was performed to assess the activity of cancer-related signaling pathways (29). The results indicated that Cluster 2 exhibited significantly higher activity in the Estrogen, Hypoxia, MAPK, NF-κB, p53, TNFα, and WNT signaling pathways, whereas Cluster 1 showed relatively higher activity in the VEGF signaling pathway (**Supplementary Figures 3C, D**).

### 3.4 Construction of the 4-gene SHRPI and evaluation of its prognostic significance

**Figure 3:**
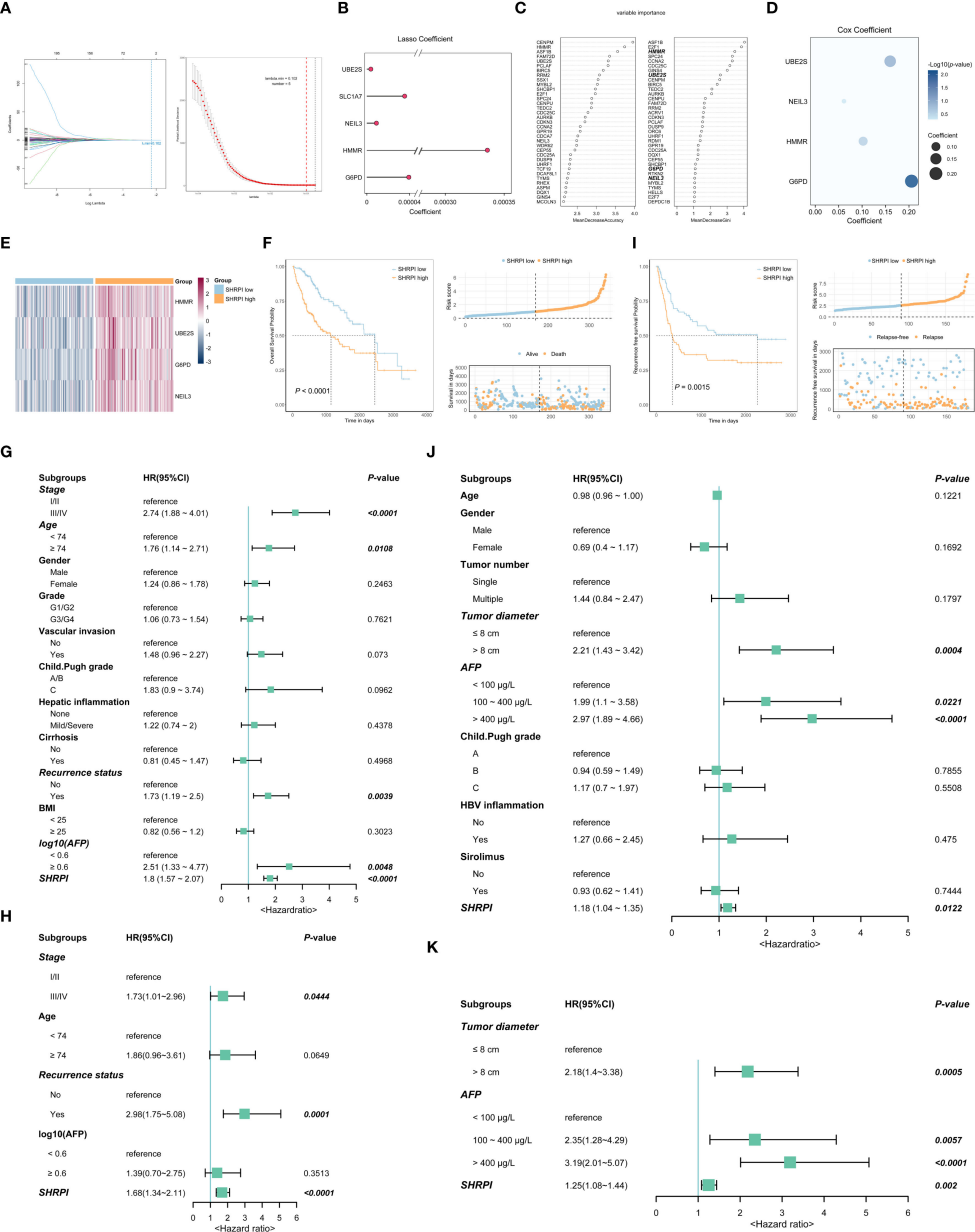
Construction of the 4-gene SHRPI and its prognostic significance. **(A)** LASSO regression analysis selected 5 variables based on the optimal lambda value. **(B)** LASSO coefficient plot for the 5 selected key genes and their coefficients. **(C)** Screening of candidate genes via random forest models. **(D)** Multivariate Cox regression analysis of the 4 selected genes used to construct the SHRPI. **(E)** Heatmap showing the expression of 4 SHRPI-related genes in low- and high-SHRPI groups. **(F)** Kaplan-Meier survival curve for OS, risk score distribution, and survival status of patients in low- and high-SHRPI groups of the TCGA-LIHC cohort. **(G, H)** Univariate **(G)** and multivariate **(H)** Cox regression analyses of SHRPI and clinicopathological parameters for OS in the TCGA-LIHC cohort. **(I)** Kaplan-Meier survival curve for RFS, risk score distribution, and relapse status of patients in low- and high-SHRPI groups of the NC-LT cohort. **(J, K)** Univariate **(J)** and multivariate **(K)** Cox regression analyses of SHRPI and clinicopathological parameters for RFS in the NC-LT cohort.

Cox regression analysis was conducted on the DEGs between the two clusters to identify prognostic genes. To mitigate collinearity effects, genes with a Pearson correlation coefficient greater than 0.80 were excluded, resulting in 221 DEGs (**Supplementary Table 2**). LASSO regression analysis was then applied, yielding five stable prognostic genes (**Figures 3A, B**). To further reduce false-positive rates and enhance model accuracy, we employed a random forest model to select genes with a Mean Decrease Gini greater than 1 (**Figure 3C**). Subsequently, four overlapping key genes were incorporated into a Cox regression model, with corresponding coefficients used to construct the SHRPI (**Figure 3D**): SHRPI = 0.10211669 × HMMR + 0.15981839 × UBE2S + 0.20537184 × G6PD + 0.06132584 × NEIL3.

Patients were stratified into low- and high-SHRPI groups based on the median SHRPI score. Differential expression analysis revealed that all four key genes were upregulated in the high-SHRPI group (**Figure 3E**; **Supplementary Figures 4A, B**). In the TCGA-LIHC cohort, patients in the high-SHRPI group exhibited significantly shorter OS compared to those in the low-SHRPI group (*P* < 0.0001) (**Figure 3F**). Time-dependent ROC curve analysis demonstrated that SHRPI exhibited robust and stable predictive performance for survival, with AUC values of 0.82, 0.70, and 0.67 for 1-, 3-, and 5-year OS, respectively (**Supplementary Figure 4C**). Univariate and multivariate Cox regression analyses confirmed that stage, recurrence status, and SHRPI were independent risk factors for OS in HCC (**Figures 3G, H**). In the NC-LT cohort, patients in the high-SHRPI group exhibited significantly shorter RFS compared to those in the low-SHRPI group (*P* = 0.0015) (**Figure 3I**). Univariate and multivariate Cox regression analyses confirmed that tumor diameter, AFP levels, and SHRPI were independent risk factors for RFS in HCC after LT (**Figures 3J, K**). Furthermore, the applicability of SHRPI was validated in the GSE104580 cohort, where SHRPI was significantly higher in the TACE non-response group compared to the response group (*P* < 0.001), with an AUC of 0.713 for predicting TACE response (**Supplementary Figures 4D, E**).

### 3.5 Development and validation of the SHRPI-based nomogram for OS prediction in HCC patients

**Figure 4:**
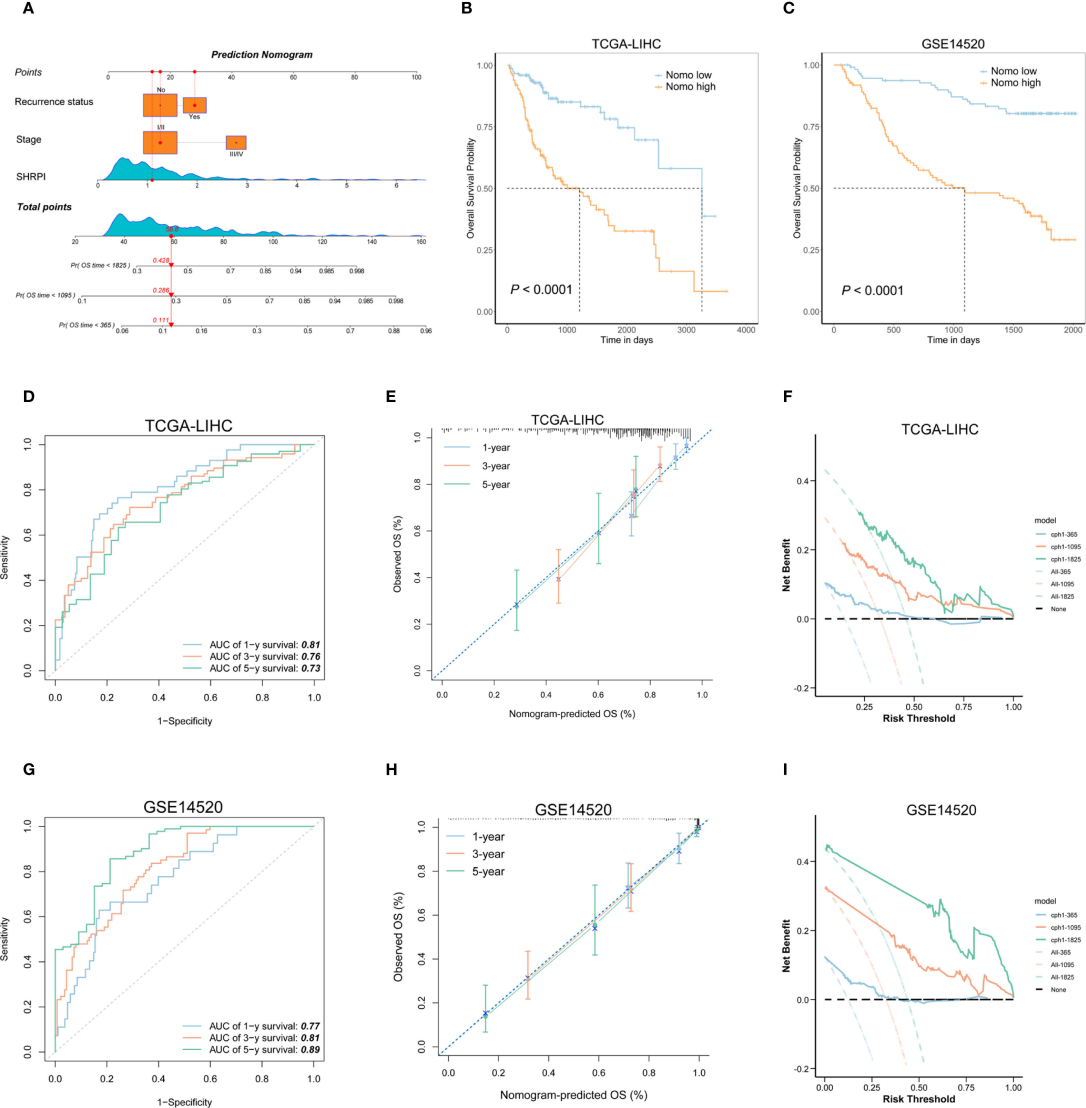
Development and validation of the SHRPI-based nomogram for OS prediction. **(A)** Nomogram for predicting OS in HCC patients, integrating SHRPI, stage, and recurrence status. **(B, C)** Kaplan-Meier survival curves comparing OS between low- and high- Nomo score groups in the TCGA-LIHC and GSE14520 cohorts. **(D, G)** Time-dependent ROC curves illustrating the nomogram’s predictive accuracy for 1-, 3-, and 5-year OS in these cohorts. **(E, H)** Calibration plots comparing predicted and observed OS at 1-, 3-, and 5-year time points across cohorts. **(F, I)** DCA demonstrating the net clinical benefit of the nomogram across different risk thresholds for OS prediction in both cohorts.

We constructed a nomogram incorporating SHRPI and other independent prognostic risk factors using the TCGA-LIHC cohort and externally validated it in the GSE14520 cohort to comprehensively assess its predictive performance (**Figure 4A**). The results demonstrated that the nomogram exhibited excellent performance in both the training and validation cohorts. After stratifying patients in both cohorts based on the median nomogram score, Kaplan-Meier survival analysis revealed that patients in the high-nomogram score group had significantly shorter OS compared to those in the low-nomogram score group (**Figures 4B, C**).

In the training cohort, the AUC for 1-, 3-, and 5-year OS was 0.81, 0.76, and 0.73, respectively, highlighting the nomogram’s strong discriminatory power for short- to medium-term survival prediction (**Figure 4D**). In the validation cohort, the nomogram maintained robust predictive performance, with AUC values of 0.77, 0.81, and 0.89 for 1-, 3-, and 5-year OS, respectively, indicating its generalizability across different datasets (**Figure 4G**). The calibration curves for both the training and validation cohorts further confirmed the accuracy of the nomogram in predicting 1-, 3-, and 5-year OS. These curves demonstrated a high degree of concordance between predicted and observed survival probabilities, underscoring the nomogram’s reliability in long-term survival estimation (**Figures 4E, H**). Additionally, DCA for both cohorts demonstrated that the nomogram yields net benefits across a broad range of risk thresholds, further supporting its potential role in guiding clinical decision-making (**Figures 4F, I**).

### 3.6 Comprehensive analysis of SHRPI and its associations with immune infiltration, TIME signatures

To further explore the biological and clinical significance of SHRPI, we calculated patient risk scores using the SHRPI formula and stratified the TCGA-LIHC cohort into low-risk (LRG) and high-risk (HRG) groups based on the optimal cutoff value. Differential expression analysis between the two groups identified key genes associated with SHRPI. Functional enrichment analyses, including GO, KEGG, GSEA, and GSVA, revealed that the majority of enriched pathways were primarily related to cell cycle regulation and metabolic processes (**Supplementary Figures 5A–D**). We performed a stemness assessment on patients in the TCGA-LIHC cohort using stemness-related gene sets from MSigDB. The Wong Embryonic Stem Cell Core score showed a significant positive correlation with the risk score, while the Yamashita Liver Cancer Stem Cell Dn score exhibited a significant negative correlation (**Supplementary Figure 5E**).

**Figure 5:**
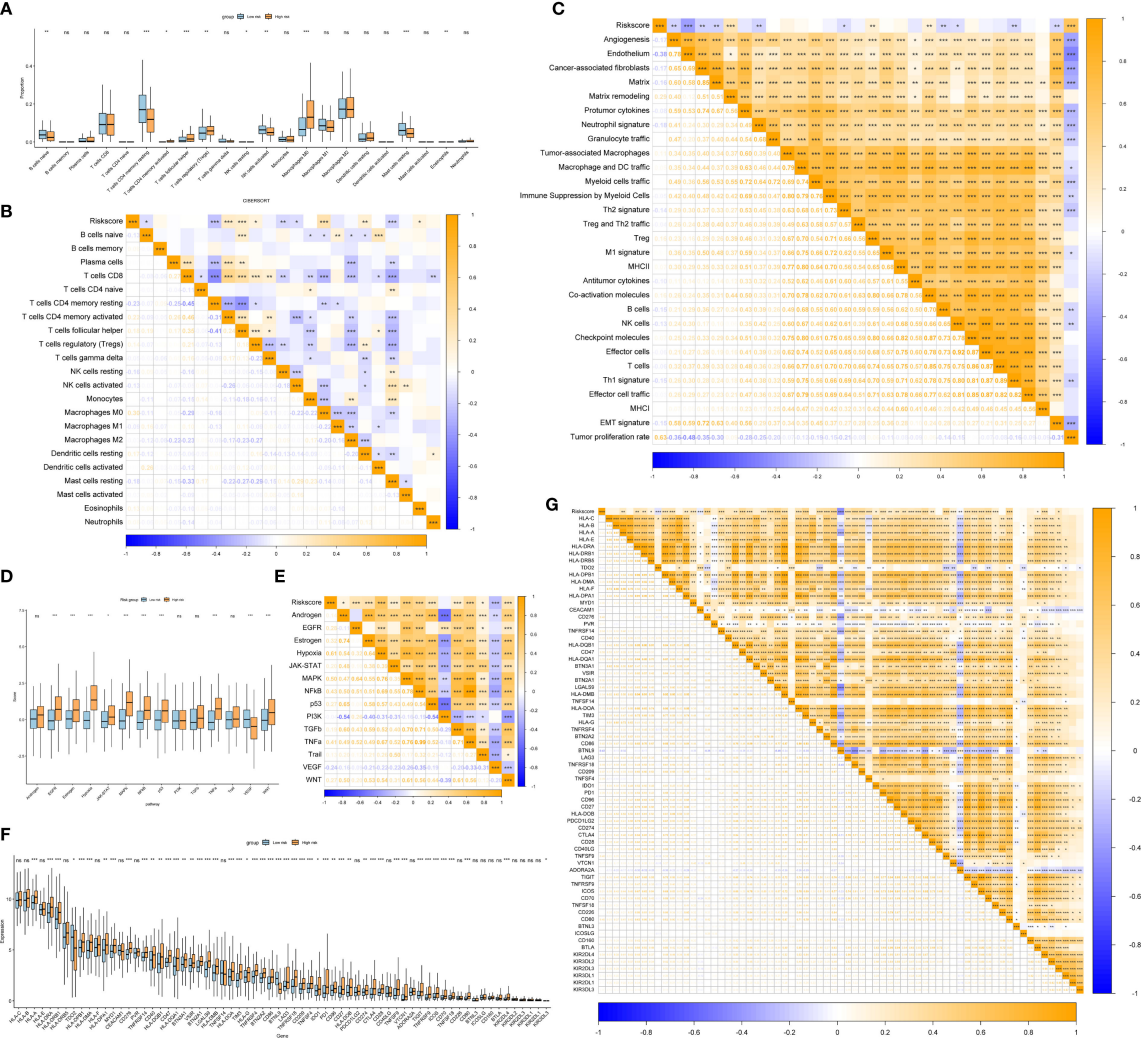
Associations of SHRPI with immune infiltration and TME signatures. **(A, B)** Comparison of immune cell infiltration levels between low- and high-risk groups **(A)** and correlation analysis of SHRPI with immune cell infiltration based on CIBERSORT **(B)**. **(C)** Correlation analysis of SHRPI with TME-related signatures. **(D, E)** Comparisons of 14 oncogenic pathways between low- and high-risk groups **(D)**, and correlation analysis of SHRPI with these pathways **(E)**. **(F, G)** Comparisons of representative immune checkpoint genes expression between low- and high-risk groups **(F)**, and correlation analysis of SHRPI with these genes **(G)**. Statistical significance: *P < 0.05, **P < 0.01, ***P < 0.001; ns = not significant.

To investigate the relationship between the risk score and immune characteristics, we analyzed the infiltration of 22 immune cell types in TIME. The results indicated a significant correlation between the risk score and multiple immune cell subsets, with distinct infiltration patterns observed between LRG and HRG, consistent with the trends observed in the stemness-hypoxia-based clusters. Specifically, HRG patients exhibited increased Tregs, follicular helper T cells (Tfh), and M0 macrophages, while resting memory CD4^+^ T cells and naïve B cells were significantly reduced (**Figures 5A, B**). Additionally, the risk score exhibited strong positive correlations with matrix remodeling, Treg abundance, and tumor proliferation rate, indicating its potential role in fostering an immunosuppressive and tumorigenic microenvironment (**Figure 5C**). The expression profiles of 68 immune checkpoint genes differed significantly between LRG and HRG, with many genes including PDCD1 (PD-1), CTLA4, CD274 (PD-L1), and HAVCR2 (TIM-3) upregulated in HRG (**Figure 5F**). Correlation analysis further revealed that the risk score was positively associated with most immune checkpoint genes, suggesting a link to an immunosuppressive tumor microenvironment and enhanced immune evasion (**Figure 5G**). Next, we assessed the potential immunotherapy responses of patients across different risk groups using TIDE scores. The results showed that in HRG, the risk score was positively correlated with T cell exclusion (ρ = 0.22, *P* = 0.02553), suggesting a higher likelihood of immune evasion. Conversely, in LRG, the risk score exhibited a stronger positive correlation with IFNG expression (ρ = 0.1984, *P* = 0.002146), indicating a potentially enhanced anti-tumor immune response despite a higher correlation with T cell exclusion (ρ = 0.3204, *P* < 0.0001) (**Supplementary Figure 5F**). Collectively, these findings suggest that HRG is characterized by a more immunosuppressive tumor microenvironment, whereas LRG retains relatively higher immune activity, which may contribute to better immunotherapy responsiveness. Finally, we analyzed the relationship between the risk score and oncogenic pathway activity. The results showed that the risk score was significantly associated with the activation of hypoxia, MAPK, NF-κB, p53, TNFα, and WNT signaling, all of which were upregulated in HRG (**Figures 5D, E**).

### 3.7 Identification of potential therapeutic agents for HRG

**Figure 6:**
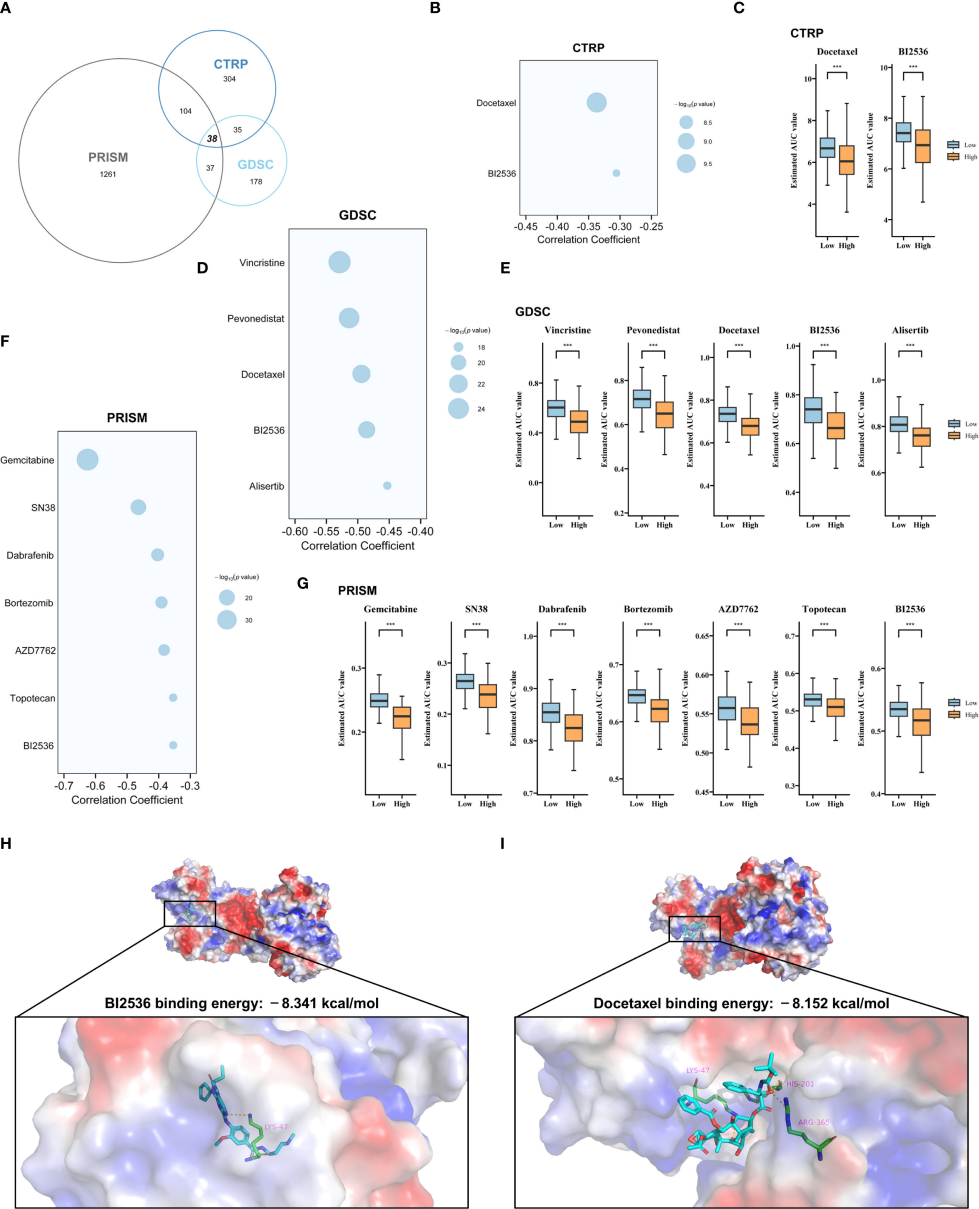
Screening of potential therapeutic agents for HRG and molecular docking analysis. **(A)** Overlapping compounds in CTRP, GDSC and PRISM datasets. **(B–G)** Spearman correlation between SHRPI and compound sensitivity, and differential drug responses (AUC) between low- and high-risk groups based on CTRP **(B, C)**, GDSC **(D, E)**, and PRISM **(F, G)** databases. **(H, I)** Molecular docking analysis of G6PD protein with two selected compounds: BI2536 **(H)** and Docetaxel **(I)**. Statistical significance: ***P < 0.001.

Sensitivity analysis revealed that conventional chemotherapeutic agents and inhibitors targeting FGFR, EGFR, and VEGFR exhibited no significant specificity in HRG. (**Supplementary Figure 6A–D**). To identify potential therapeutic agents with greater efficacy in HRG, we leveraged the CTRP, PRISM, and GDSC datasets, which provide comprehensive gene expression and drug sensitivity profiles across hundreds of human cancer cell lines. Following the removal of duplicates and quality control, 38 candidate compounds were selected for further evaluation (**Figure 6A**). Initially, compounds with lower estimated AUC values in HRG were identified using predefined thresholds (log2FC < −0.1 for CTRP and GDSC, and log2FC < −0.05 for PRISM). Subsequently, Spearman correlation analysis was performed to assess the association between AUC values and the risk score, with further filtering applied to compounds exhibiting a negative correlation (R < −0.3 for CTRP and PRISM, and R < −0.4 for GDSC) (**Figures 6B, D, F**). Ultimately, we identified 2 compounds from CTRP (docetaxel, BI2536), 5 from GDSC (vincristine, pevonedistat, docetaxel, BI2536, alisertib), and 7 from PRISM (gemcitabine, SN38, dabrafenib, bortezomib, AZD7762, topotecan, BI2536), all of which demonstrated lower estimated AUC values in HRG, indicating greater predicted sensitivity to these agents (**Figures 6C, E, G**). Given that G6PD was the molecule most closely associated with prognosis in Cox regression analysis among the components of SHRPI (**Figure 3D**), compounds identified in at least two database screens (Docetaxel and BI2536) were selected for molecular docking with G6PD. The results demonstrated that BI2536 had a higher binding affinity for G6PD (−8.341 kcal/mol) compared to Docetaxel (−8.152 kcal/mol) (**Figures 6H, I**). These findings indicate that BI2536 may regulate G6PD activity by directly targeting its active site, thereby potentially influencing HCC stemness.

### 3.8 Analysis and functional validation of G6PD as a key SHRPI component in HCC

**Figure 7:**
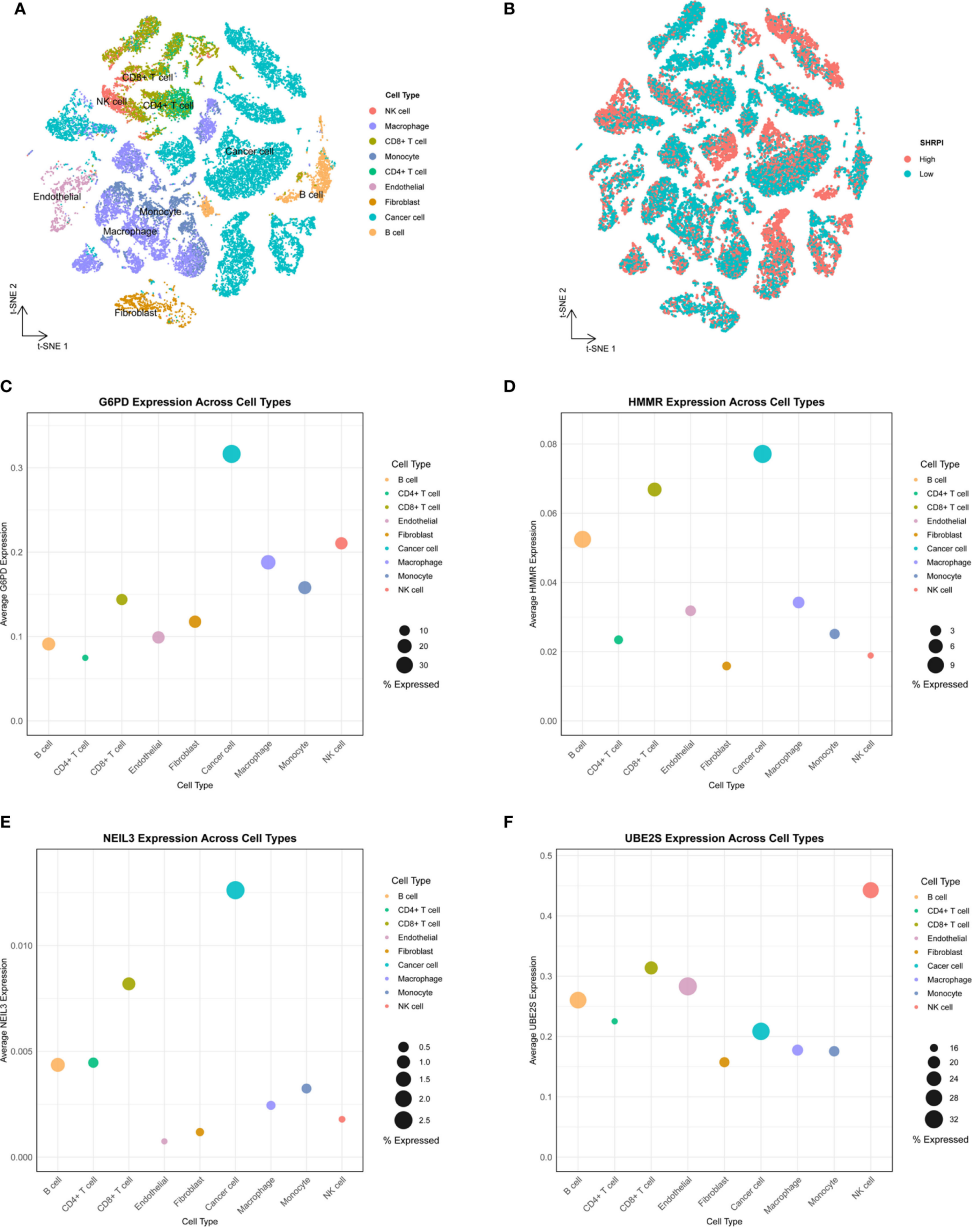
Single‐cell transcriptomic analysis of SHRPI in HCC. **(A)** t-SNE plot illustrating the clustering of single cells from HCC tissues, annotated by cell type. **(B)** t-SNE plot showing the distribution of SHRPI status (high *vs*. low). **(C–F)** Average expression levels of G6PD **(C)**, HMMR **(D)**, NEIL3 **(E)**, and UBE2S **(F)** across cell types.

**Figure 8:**
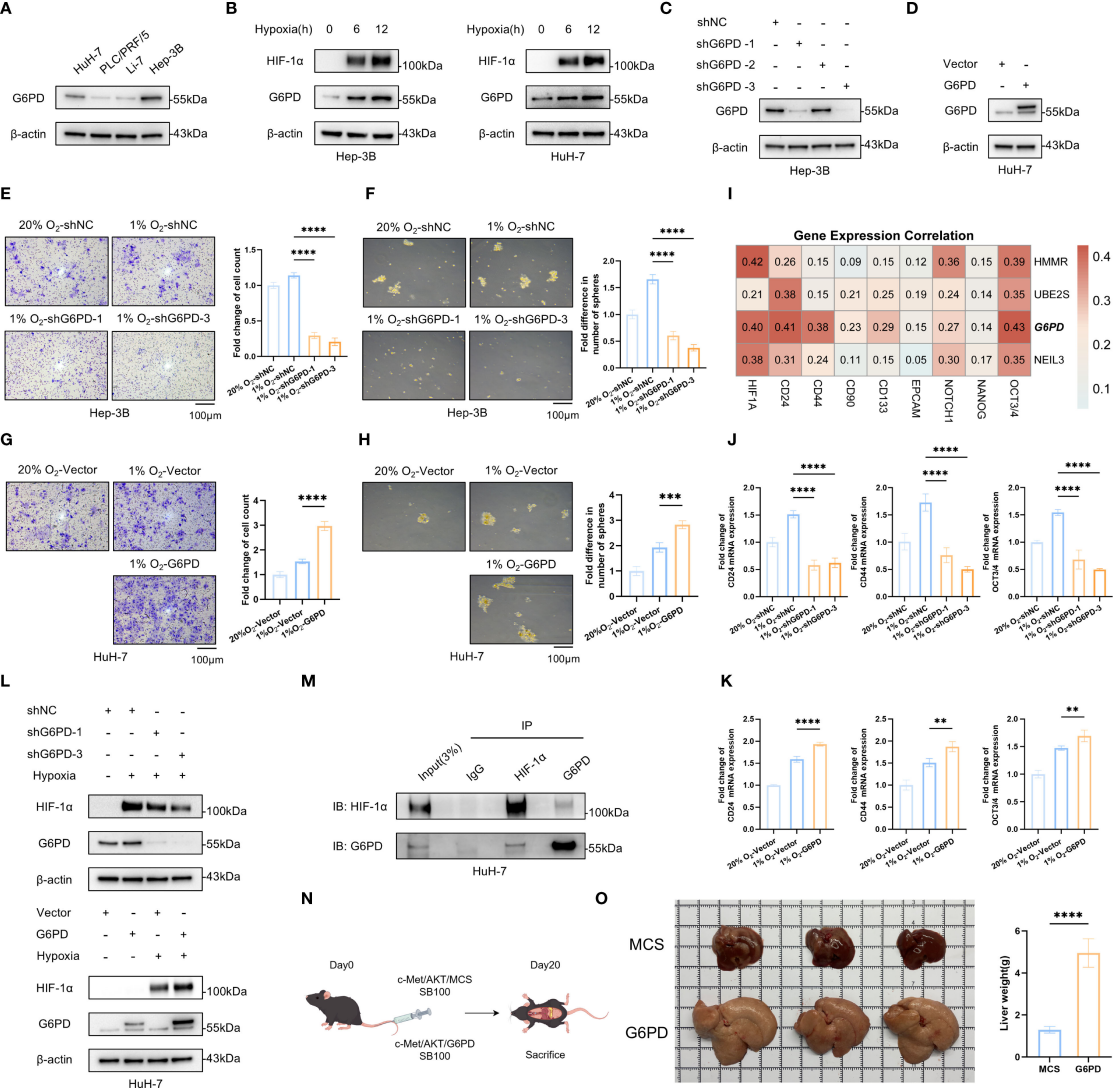
Functional validation of G6PD as a key SHRPI component in HCC. **(A)** G6PD protein abundances across HCC cell lines (HuH-7, PLC/PRF/5, Li-7 and Hep-3B). **(B)** Changes in HIF-1α and G6PD expression in HuH-7 and Hep-3B cells under normoxia (20% O_2_) or hypoxia(1%O_2_) at 6 and 12 hours. **(C)** Knockdown efficiency of three G6PD shRNAs in Hep-3B cells. **(D)** G6PD overexpression efficiency in HuH-7 cells. **(E, F, J)** Effects of shNC, shG6PD-1, and shG6PD-3 on Hep-3B stemness under hypoxia, as assessed by cell migration **(E)**, cell sphere formation **(F)**, and qRT-PCR **(J)** assays. **(G, H, K)** Effects of Vector and G6PD overexpression (G6PD) on HuH-7 stemness under hypoxia, as assessed by cell migration **(G)**, cell sphere formation **(H)**, and qRT-PCR **(K)** assays. **(I)** Correlation heatmap of SHRPI components and stemness markers in TCGA-LIHC bulk RNA-seq data. **(L)** G6PD and HIF-1α expression in HuH-7 with G6PD knockdown (shG6PD) or control (shNC) (upper panel), and with G6PD overexpression (G6PD) or Vector (lower panel), under normoxia or hypoxia. **(M)** Interaction between endogenous G6PD and HIF-1α was tested in HuH-7 under hypoxia for 24 hours, with normal rabbit IgG as control. **(N)** Experimental workflow of the transgenic HCC mouse model (by figdraw.com, ID: ARUWT24438). **(O)** Representative liver images of MCS and G6PD groups, and comparison of liver weights between the two groups (n = 5). Statistical significance: **P < 0.01, ***P < 0.001, ****P < 0.0001.

To further explore the cellular heterogeneity and expression profiles of SHRPI components in HCC, we analyzed single-cell RNA sequencing data. t-SNE dimensionality reduction and SHRPI scoring demonstrated that cancer cells exhibited significantly higher SHRPI levels (**Figures 7A, B**). Among the SHRPI components, G6PD, HMMR, and NEIL3 were predominantly expressed in cancer cells, with G6PD showing the highest expression proportion (**Figures 7C–F**). This further underscored the pivotal role of G6PD in regulating HCC stemness at the single-cell level. Upon this finding, we further investigated the changes in G6PD expression under hypoxia and its impact on the stemness phenotype in HCC cells. We first assessed G6PD expression across HCC cell lines (HuH-7, PLC/PRF/5, Hep-3B, and Li-7) via Western blot. Based on strong expression in Hep-3B and moderate expression in HuH-7, we selected these two cell lines for further experiments (**Figure 8A**). Exposure of these cells to hypoxia (1% O_2_) significantly upregulated the expression of G6PD (**Figure 8B**). To further assess the role of G6PD in regulating the stemness phenotypes of HCC cells under hypoxia, we conducted knockdown and overexpression studies. In Hep-3B cells, shRNA1 and shRNA3 achieved effective G6PD knockdown (**Figure 8C**), while in HuH-7 cells, effective overexpression was achieved (**Figure 8D**). Correlation analysis between G6PD and stemness markers showed that its expression was relatively strongly correlated with CD24, CD44, OCT3/4, and HIF-1α (**Figure 8I**). Functional assays indicated that G6PD knockdown significantly reduced cell migration, sphere formation and the mRNA expression of stemness markers (CD24, CD44, and OCT3/4) under hypoxia (**Figures 8E, F, J**). Conversely, G6PD overexpression enhanced these capabilities (**Figures 8G, H, K**). Mechanistically, we found that G6PD regulated the protein abundance of HIF-1α. In HuH-7 cells, G6PD knockdown reduced HIF-1α protein abundance, while overexpression increased HIF-1α protein abundance under hypoxia (**Figure 8L**). Co-IP experiments further confirmed the interaction between endogenous G6PD and HIF-1α in HuH-7 cells under hypoxia (**Figure 8M**). Furthermore, in the transgenic HCC mouse model, compared with the empty vector control group (MCS), mice in the G6PD overexpression group (G6PD) exhibited a rapid increase in hepatic tumor burden within a short period, as evidenced by a significant elevation in liver weight (*P* < 0.0001) (**Figures 8N, O**). In conclusion, our data indicate that G6PD is a key regulator of the stemness phenotype in HCC cells under hypoxia by interacting with HIF-1α to upregulate its protein abundance, thereby promoting the stemness phenotype.

## 4 Discussion

The collaborative crosstalk between cancer stemness and hypoxia in HCC highlights their critical roles as drivers of tumor invasion, metastatic dissemination, therapy resistance, and immune escape mechanisms. To quantify these two biological characteristics, Malta et al. developed the OCLR algorithm, which calculates stemness indices (e.g., mRNAsi, mDNAsi) by integrating multi-omics data spanning genomic, transcriptomic, and epigenetic features (25). Similarly, hypoxia scores such as the Buffa Hypoxia Score and Winter Hypoxia Score, derived from gene expression signatures, reflect molecular adaptations to oxygen deprivation (39). Although some of these indices independently correlate with adverse HCC prognosis, current prognostic models often either treat stemness and hypoxia as isolated biological characteristics, thereby neglecting their dynamic crosstalk, or dissociate these axes from clinical parameters, consequently diminishing predictive accuracy and clinical applicability (18, 19, 21). This notable gap underscores the necessity to develop integrated indices bridging these axes (stemness and hypoxia) for refined risk stratification and prioritized therapeutic targeting, while incorporating clinical parameters to optimize predictive performance through a holistic representation of HCC biology.

In this study, we selected the stemness index (mRNAsi) and the hypoxia score (Buffa hypoxia score), both of which are significantly associated with the prognosis of HCC patients. Through differential analysis and WGCNA, we identified SHRGs that comprehensively represent the interaction between stemness and hypoxia. Consensus clustering based on SHRGs stratified HCC patients into two distinct clusters (Cluster 1/2) with divergent genomic alterations, intrinsic immunogenicity, and survival outcomes. Notably, the enrichment of oncogenic pathways (e.g., E2F targets, mTOR signaling) and elevated TMB in Cluster 2 suggested heightened genomic instability and therapeutic resistance, consistent with observations in other solid tumors (40, 41). Importantly, Cluster 2 patients displayed elevated TIDE scores and upregulated immune checkpoint molecules (PD-L1, CTLA4), which extends the utility of our stemness-hypoxia signature to predict immunotherapy response, a dimension that has been underexplored in earlier studies.

Given that previous studies predominantly relied on Cox regression or heuristic gene screening to construct indices, we integrated LASSO regression, random forest, and Cox regression analysis to minimize overfitting risks while prioritizing biologically relevant key genes. This optimized rigorous strategy constructed the SHRPI comprising four genes: HMMR, UBE2S, NEIL3, and G6PD. SHRPI is not only an independent risk factor for OS in HCC patients but also for RFS in LT patients with HCC beyond the Milan criteria. Additionally, as stemness-hypoxia features are frequently associated with TACE treatment in advanced HCC, we observed that SHRPI demonstrated promising predictive power for TACE responsiveness (42). Incorporating SHRPI into a nomogram further enhanced its clinical utility, enabling personalized survival probability assessments. This approach contrasts with previous studies that focused solely on risk stratification based on indices. The nomogram exhibited robust performance in both TCGA and GSE14520 cohorts, highlighting its broad applicability.

Analysis of the TIME revealed significant disparities between risk groups defined by SHRPI. High-risk patients exhibited heightened infiltration of immunosuppressive cells (e.g., Tregs, M0 macrophages) and elevated expression of checkpoint molecules (PD-1, CTLA-4, TIM-3), consistent with the “immune-excluded” phenotype observed in CSC-enriched tumors (4, 43). SHRPI demonstrated a positive correlation with TIDE scores, indicating limited efficacy of immune checkpoint inhibitor (ICI) therapy in high-risk patients, necessitating exploration of alternative or combinatory therapeutic strategies. To address this, we screened for subgroup-specific therapeutic agents using pharmacogenomics and molecular docking analyses. Among these, BI2536 stood out due to its consistent validation across three independent datasets and strong binding affinity, highlighting its potential as a candidate drug for high-risk HCC. As a specific inhibitor of Polo-like kinase 1 (PLK1), BI2536 synergizes with diverse chemotherapies (e.g., microtubule-targeting agents, alkylators, platinum drugs) across multiple preclinical cancer models, enhancing tumor suppression and overcoming chemoresistance by inducing G2/M arrest, activating apoptosis via BAX/caspase-3 pathways and pyroptosis via GSDME, and modulating critical signaling cascades (Wnt/β-catenin, MEK/ERK). In the targeted therapy domain, BI2536 demonstrates synergistic efficacy against ROCK, mTOR, STAT3, EGFR, PARP, HDAC, and Bcr-Abl, overcoming both intrinsic and acquired resistance through dual-pathway blockade, restoration of tumor suppressor function (e.g., TP53 reactivation), and enhancement of DNA damage responses (44). Regarding immunotherapy, although clinical evidence for BI2536 combination remains scarce, emerging preclinical insights indicate that PLK1 inhibition broadly potentiates antitumor immunity through enhanced antigen presentation and T-cell infiltration, reversal of immunosuppressive TAM polarization (from M2 to M1 phenotype), and upregulation of PD-L1 expression via the PLK1/Rb/NF-κB axis to sensitize tumors to immune checkpoint blockade (45). Given that BI2536’s limited single-agent efficacy and dose-limiting toxicities indicate intrinsic resistance, CRISPR/Cas9 genome-wide screening to identify resistance genes and delineate BI2536-specific pathways is essential for optimizing pharmacological properties, enhancing therapeutic efficacy in rational combination regimens, and advancing clinical translation (46).

Among the four hub genes, hyaluronan-mediated motility receptor (HMMR/RHAMM) is highly expressed in lung, breast, gastric, and liver cancers, correlating with poor prognosis (47). HMMR critically maintains cancer stemness across tumor types: sustaining glioblastoma stem cell tumorigenicity (48), enhancing gastric cancer stemness and 5-fluorouracil resistance via TGF-β/Smad2 (49), and promoting glycolysis to strengthen stemness and cisplatin resistance in lung adenocarcinoma (50). Although direct evidence linking HMMR to hypoxia regulation is limited, hypoxia may indirectly potentiate HMMR’s oncogenic effects by activating key stemness-associated pathways, notably TGF-β signaling and glycolysis, both established hypoxia-responsive processes (51). Furthermore, HMMR predicts immunosuppressive microenvironments in HCC, with its targeting enhancing anti-PD-1 efficacy through CD8^+^ T cell recruitment (52). Ubiquitin-conjugating enzyme E2S (UBE2S), a crucial member of the ubiquitin-proteasome system, is overexpressed in multiple cancers (e.g., lung, bladder, ovarian, liver) and correlates with poor prognosis and advanced stage. UBE2S promotes cancer stemness through diverse mechanisms: enhancing p53 ubiquitination to facilitate proliferation and migration, accelerating cell cycle progression via p27 ubiquitination, and inducing chemoresistance through both the PTEN/AKT and Wnt/β-catenin signaling pathways (53). Notably, UBE2S directly ubiquitinates VHL independent of canonical E3 ligases, regulating HIF-1α signaling to promote glycolysis and HCC proliferation (54). It further drives tumor growth and reduces sorafenib sensitivity by upregulating HIF-1α and activating JAK2/STAT3 signaling (55). These findings suggest that UBE2S may function as a molecular bridge linking stemness and hypoxia regulation in HCC. Nei endonuclease VIII-like 3 (NEIL3), a DNA glycosylase crucial for repairing oxidative DNA damage and crosslinks, is highly expressed in multiple cancers (e.g., lung, kidney, liver) and promotes tumor progression. In HCC, NEIL3 not only repairs telomeric oxidative damage to delay cellular senescence but also activates the BRAF/MEK/ERK/TWIST pathway to induce core stemness phenotypes including epithelial-mesenchymal transition, therapy resistance, and enhanced self-renewal (56). Concurrently, it remodels metabolic microenvironments via MAZ-mediated aerobic glycolysis to support stemness maintenance (57), while driving malignant expansion of CSCs through the SNHG3/E2F1 axis (58). Although direct links to hypoxia regulation remain limited, a defined mechanism shows that NEIL3 enables proper expression of hypoxia-responsive genes by repairing hypoxia-associated oxidative damage in promoter G-quadruplex DNA, leading to reduced genomic instability under hypoxia (59).

Glucose-6-phosphate dehydrogenase (G6PD), the rate-limiting enzyme of the pentose phosphate pathway (PPP), maintains redox homeostasis through NADPH generation and supports nucleotide biosynthesis via ribose-5-phosphate production. Clinically significant overexpression of G6PD has been documented in multiple malignancies, including lung, renal, breast and liver cancer, and it correlates with adverse clinical outcomes (60). Hypoxic tumor microenvironments in HCC induce transcriptional upregulation of G6PD, which confers survival advantages through oxidative stress modulation (61). Emerging evidence demonstrates that G6PD overexpression diminishes regorafenib cytotoxicity in HCC (62), while METTL3-mediated activation of G6PD-dependent PPP flux drives oxaliplatin resistance (63). Therefore, the dual regulatory role of G6PD in maintaining redox equilibrium and facilitating metabolic reprogramming serves as a critical determinant in preserving cancer cell stemness under various stress conditions. Integrating multivariate Cox regression and single-cell transcriptomic analyses based on SHRPI components identified G6PD as a key prognostic determinant closely associated with HCC stemness regulation at the single-cell level. Its specific role in hypoxia-driven stemness maintenance has yet to be reported. Through systematic experiments, we demonstrated that hypoxia significantly upregulates G6PD expression in HCC cells and that G6PD is essential for maintaining cancer stemness. Mechanistically, we showed that G6PD stabilizes HIF-1α protein under hypoxia. Given established evidence that HIF-1α transcriptionally activates G6PD (61, 64), we propose a self-reinforcing positive feedback loop that amplifies HCC stemness. Within this regulatory mechanism, G6PD may enhance HIF-1α stability through dual mechanisms: as a core metabolic enzyme, it potentially modulates redox homeostasis to attenuate degradation (65); while exercising non-canonical enzymatic functions, it may directly suppress HIF-1α ubiquitination (66). Endogenous Co-IP confirmed G6PD-HIF-1α interaction, thereby providing mechanistic support for these stabilization pathways. These findings reveal a novel metabolic-microenvironmental crosstalk driving stemness; this manifestation of non-canonical molecular functions bridging metabolism and TME remodeling is similarly observed in recent biomarker studies of other solid tumors (67). Collectively, they suggest that targeting the G6PD-HIF-1α loop aligns with the emerging paradigm of combinatorial strategies against multiple tumor microenvironment components (68) and provide both novel insights and a theoretical foundation for therapeutic strategies aimed at targeting cancer stemness.

Although previous studies incorporated stemness or hypoxia characteristics for HCC prognostication, our work delivers substantial advances: algorithmic refinement optimizes SHRPI to identify a minimal gene set with maximal prognostic power, significantly enhancing discriminative performance and model parsimony; we develop a highly accurate and clinically applicable nomogram for individualized prognosis; pharmacogenomic and molecular docking analyses rigorously screened BI2536 as a promising agent for the high-risk subgroup, providing actionable insights for therapeutic stratification; integrating computational and experimental evidence, we first establish G6PD as a key regulator of hypoxia-induced stemness and propose a G6PD-HIF-1α positive feedback loop as a mechanistic model.

Despite these advancements, our study has limitations. First, given the retrospective nature and potential ethnic composition bias of TCGA/GEO data, prospective validation of SHRPI in multi-ethnic cohorts is necessary to assess its applicability, and its predictive capacity for immunotherapy response requires further validation in diverse immunotherapy cohorts (69). Second, deeper molecular investigations are required to elucidate the specific regulatory mechanisms of G6PD on HIF-1α, alongside comprehensive spatial single-cell analyses and *in vivo* lineage tracing to resolve spatial heterogeneity in G6PD-HIF-1α interactions within tumor microenvironments. Third, preclinical assessment of candidate compounds (BI2536) using patient-derived organoids or xenograft models should systematically evaluate on-target efficacy versus off-target toxicity profiles to ensure safety of combinatorial regimens for high-risk populations (70).

## 5 Conclusions

SHRPI, constructed using a rigorous methodological approach, effectively distinguishes clinical, molecular, TIME, and therapeutic response characteristics among HCC patients. The nomogram integrating SHRPI with key clinical parameters demonstrates high predictive accuracy and robust applicability. BI2536 showed promising therapeutic potential for patients classified as high-risk by SHRPI. Furthermore, the elucidation of the hypoxia-stemness regulatory mechanism mediated by G6PD, a key SHRPI component, provides novel insights into therapeutic strategies targeting HCC stemness.

## Data Availability

The datasets generated during and/or analysed during the current study are available in the following repositories: TCGA (https://www.cancer.gov/ccg/research/genome-sequencing/tcga), GEO (https://www.ncbi.nlm.nih.gov/geo/), The cBio Cancer Genomics Portal (http://cbioportal.org), PubChem Compound (https://pubchem.ncbi.nlm.nih.gov/), PDB (http://www.rcsb.org/), and the Genome Sequence Archive database (accession codes: HRA010539, HRA010528; https://ngdc.cncb.ac.cn/). Computational scripts generated during this research are available upon formal request to the corresponding authors.

## Supplementary material

Supplementary Figure 1 Prognostic and clinical implications of stemness indices and hypoxia scores in HCC.

Supplementary Figure 2 Genomic characteristics of Cluster 1 and Cluster 2.

Supplementary Figure 3 Therapeutic and immune characteristics of Cluster 1 and Cluster 2.

Supplementary Figure 4 Predictive performance of SHRPI for OS and TACE response.

Supplementary Figure 5 Differential analysis, functional enrichment, and correlation with key gene signatures between low- and high-risk groups.

Supplementary Figure 6 Drug sensitivity comparisons between low- and high-risk groups.

Supplementary Table 1 Stemness- and hypoxia-related genes.

Supplementary Table 2 Cox regression results of DEGs after collinearity filtering for SHRPI construction.

Supplementary Table 3 Detailed information of oligonucleotides used in experiments.

Supplementary Table 4 Detailed information of antibodies used in experiments.

Supplementary Table 5 Detailed information of primers used in experiments.
