# Supplementary material for: Stemness- and hypoxia-based prognostic stratification index reveals G6PD as a regulator of hypoxia-driven stemness in hepatocellular carcinoma
Source: Front Immunol. 2025 Sep 19;16:1669275. doi: 10.3389/fimmu.2025.1669275 (PMC12491235; doi:10.3389/fimmu.2025.1669275)
Supplement: Supplementary Figure 1 — Prognostic and clinical implications of stemness indices and hypoxia scores in HCC. [file Image1.pdf]

Supplementary Material

Supplementary Figures

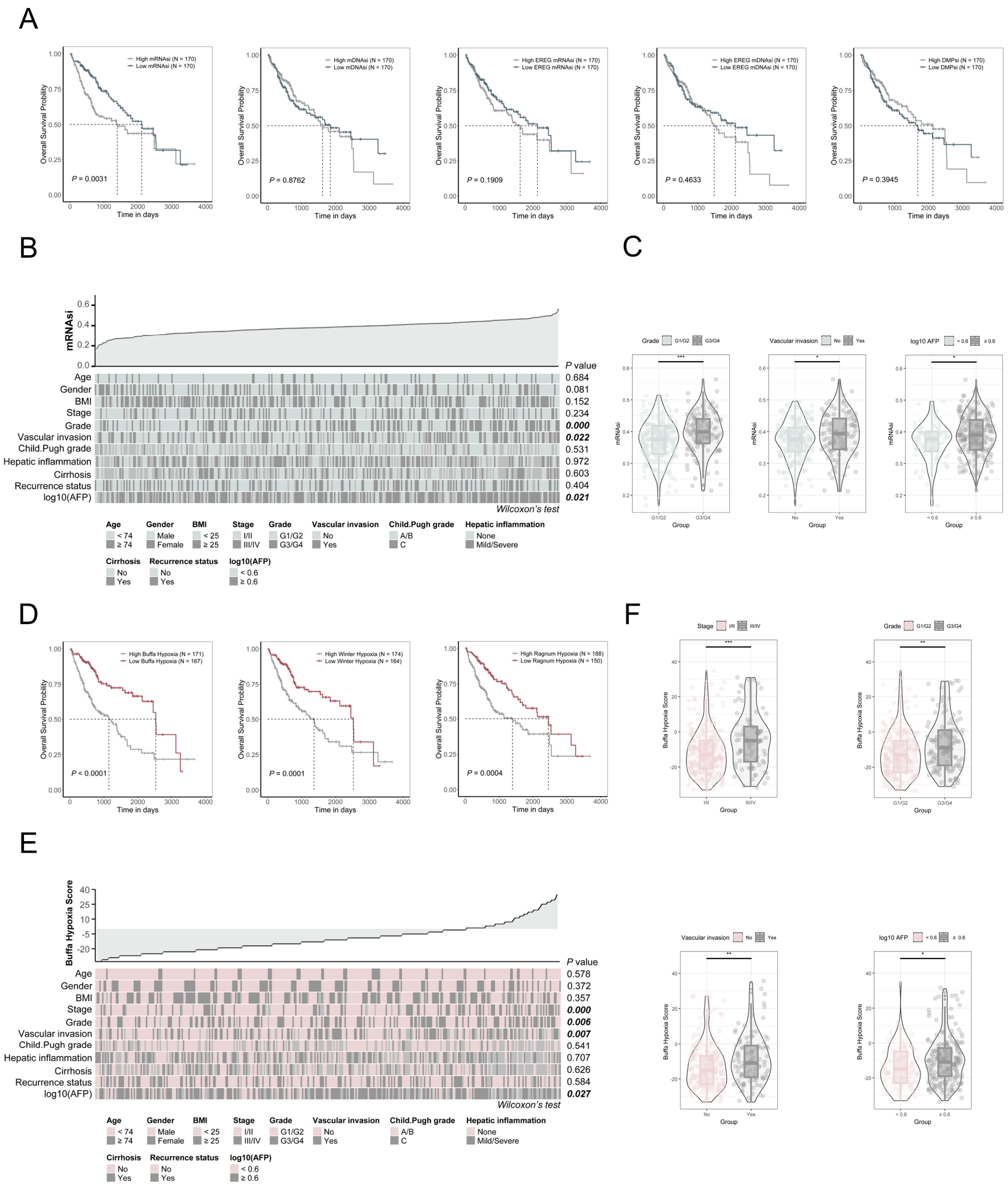

Supplementary Figure 1. Prognostic and clinical implications of stemness indices and hypoxia scores in HCC. (**A**) Kaplan-Meier survival curves for OS of HCC patients in the high and low groups of different stemness indices. (**B**) Association between mRNAsi and clinical characteristics in the TCGA-LIHC cohort. (**C**) Distribution of mRNAsi across clinical subgroups, including grade, vascular invasion, and AFP. (**D**) Kaplan-Meier survival curves for OS of HCC patients in the high and low groups of different hypoxia scores. (**E**) Association between Buffa Hypoxia Score and clinical characteristics in the TCGA-LIHC cohort. (**F**) Distribution of Buffa Hypoxia Score across clinical subgroups, including stage, grade, vascular invasion, and AFP.

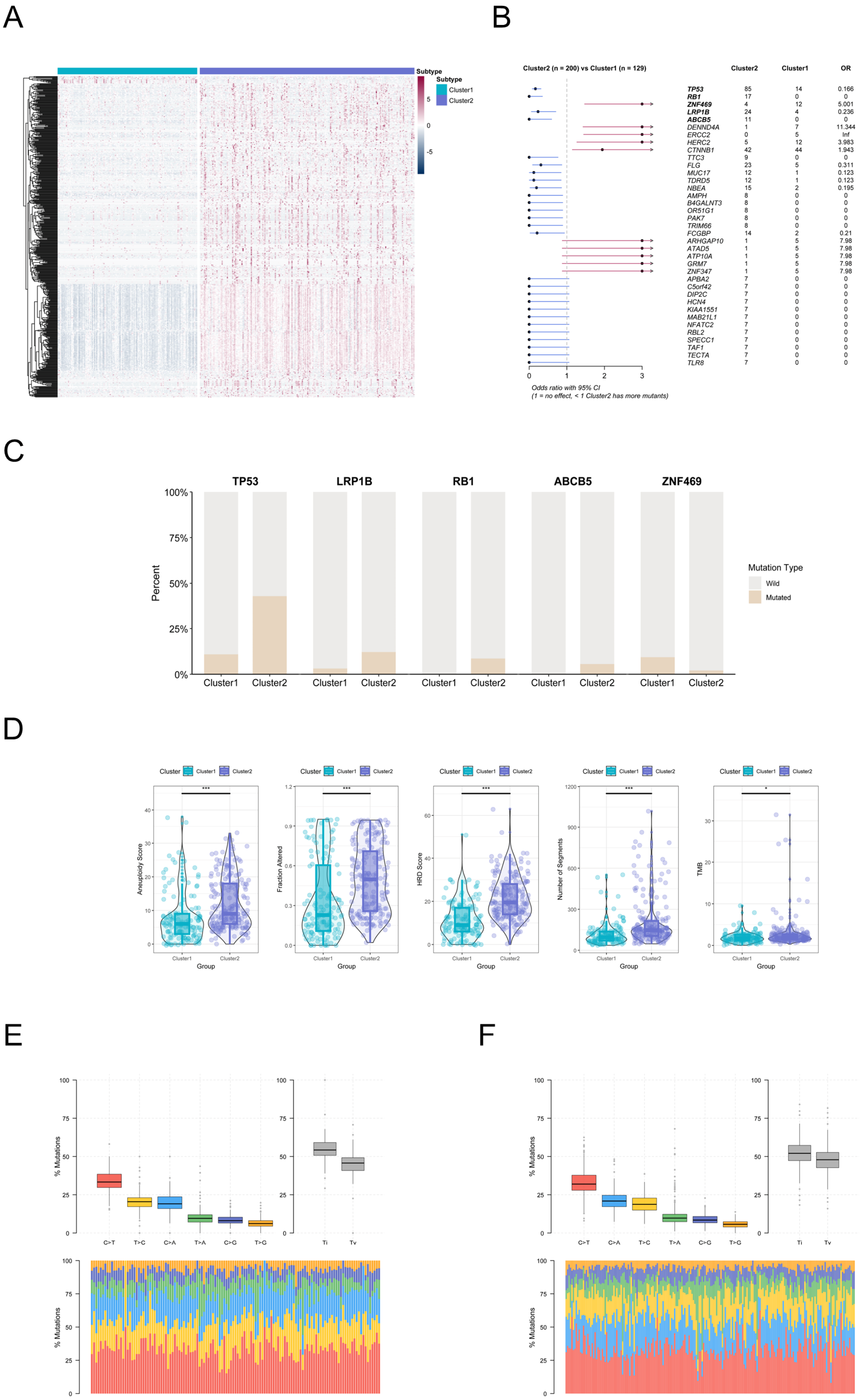
A
Subtype
Subtype
Cluster1
Cluster2
5
0
-5
B
Cluster2 (n = 200) vs Cluster1 (n = 129)
Cluster2 Cluster1 OR P-value
TP53 85 14 0.166 ***
RB1 17 0 0 ***
ZNF469 4 12 5.001 **
LRP1B 24 4 0.236 **
ABCB5 11 0 0 **
DENND4A 1 7 11.344 **
ERCC2 0 5 Inf **
HERC2 5 12 3.983 **
CTNNB1 42 44 1.943 *
TTC3 9 0 0 *
FLG 23 5 0.311 *
MUC17 12 1 0.123 *
TDRD5 12 1 0.123 *
NBEA 15 2 0.195 *
AMPH 8 0 0 *
B4GALNT3 8 0 0 *
OR51G1 8 0 0 *
PAK7 8 0 0 *
TRIM66 8 0 0 *
FCGBP 14 2 0.21 *
ARHGAP10 1 5 7.98 *
ATAD5 1 5 7.98 *
ATP10A 1 5 7.98 *
GRM7 1 5 7.98 *
ZNF347 1 5 7.98 *
APBA2 7 0 0 *
C5orf42 7 0 0 *
DIP2C 7 0 0 *
HCN4 7 0 0 *
KIAA1551 7 0 0 *
MAB21L1 7 0 0 *
NFATC2 7 0 0 *
RBL2 7 0 0 *
SPECC1 7 0 0 *
TAF1 7 0 0 *
TECTA 7 0 0 *
TLR8 7 0 0 *
0 1 2 3
Odds ratio with 95% CI
(1 = no effect, < 1 Cluster2 has more mutants)
C
TP53
LRP1B
RB1
ABCB5
ZNF469
100%
75%
50%
25%
0%
Percent
Cluster1 Cluster2
Mutation Type
Wild
Mutated
D
Cluster Cluster1 Cluster2
Aneuploidy Score
Fraction Altered
HRD Score
Number of Segments
TMB
Group
E
F
% Mutations
C>T
T>C
C>A
T>A
C>G
T>G
Ti
Tv

Supplementary Figure 2. Genomic characteristics of Cluster 1 and Cluster 2. (**A**) Heatmap showing the expression profiles of DEGs between the two clusters. (**B**) Forest plot displaying odds ratios (OR) with 95% confidence intervals for mutation frequencies of significantly mutated genes between the two clusters. (**C**) Mutation rates of representative genes in the two clusters. (**D**) Differences in Aneuploidy Score, Fraction Altered, HRD Score, Number of Segments, and TMB between the two clusters. (**E, F**) Base substitution and Ti/Tv ratio distributions of SNVs in Cluster 1 (E) and Cluster 2 (F).

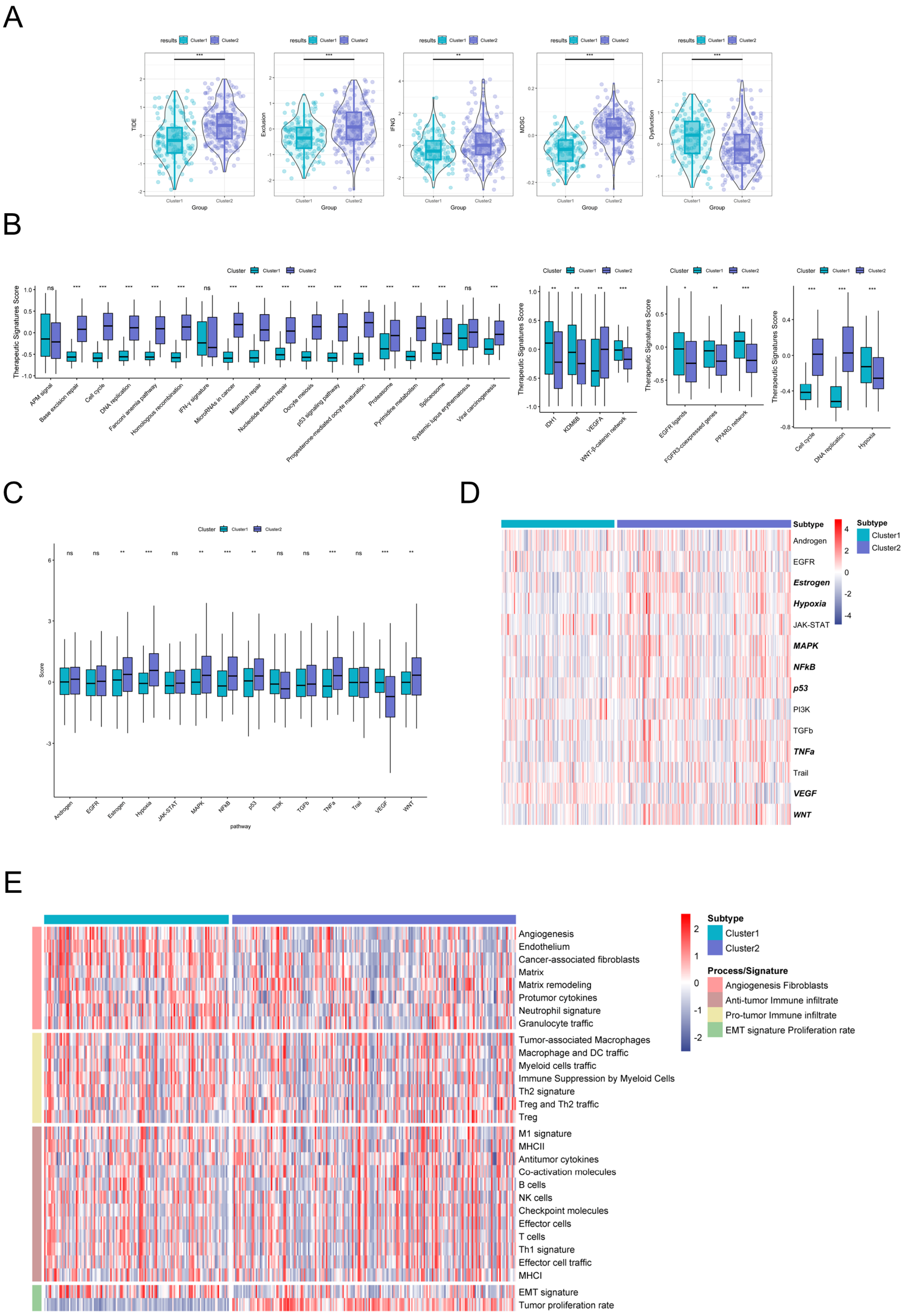
A
B
C
D
E
Cluster
Cluster1
Cluster2
Score
pathway
Androgen
EGFR
Estrogen
Hypoxia
JAK-STAT
MAPK
NFkB
p53
PI3K
TGFb
TNFa
Trail
VEGF
WNT
Subtype
Angiogenesis
Endothelium
Cancer-associated fibroblasts
Matrix
Matrix remodeling
Protumor cytokines
Neutrophil signature
Granulocyte traffic
Tumor-associated Macrophages
Macrophage and DC traffic
Myeloid cells traffic
Immune Suppression by Myeloid Cells
Th2 signature
Treg and Th2 traffic
Treg
M1 signature
MHCII
Antitumor cytokines
Co-activation molecules
B cells
NK cells
Checkpoint molecules
Effector cells
T cells
Th1 signature
Effector cell traffic
MHCI
EMT signature
Tumor proliferation rate
Process/Signature
Angiogenesis Fibroblasts
Anti-tumor Immune infiltrate
Pro-tumor Immune infiltrate
EMT signature Proliferation rate

Supplementary Figure 3. Therapeutic and immune characteristics of Cluster 1 and Cluster 2. (**A**) Differences in TIDE scores, Exclusion scores, Dysfunction scores, IFNG expression, and MDSC levels between the two clusters. (**B**) Differences in therapeutic signature scores, including hallmark pathways, DNA damage and repair pathways, and immune-related pathways, between the two clusters. (**C, D**) Comparisons of the 14 oncogenic pathways in the two clusters using the PROGENy algorithm. (**E**) Comparisons of the abundances of 29 gene signatures in the two clusters.

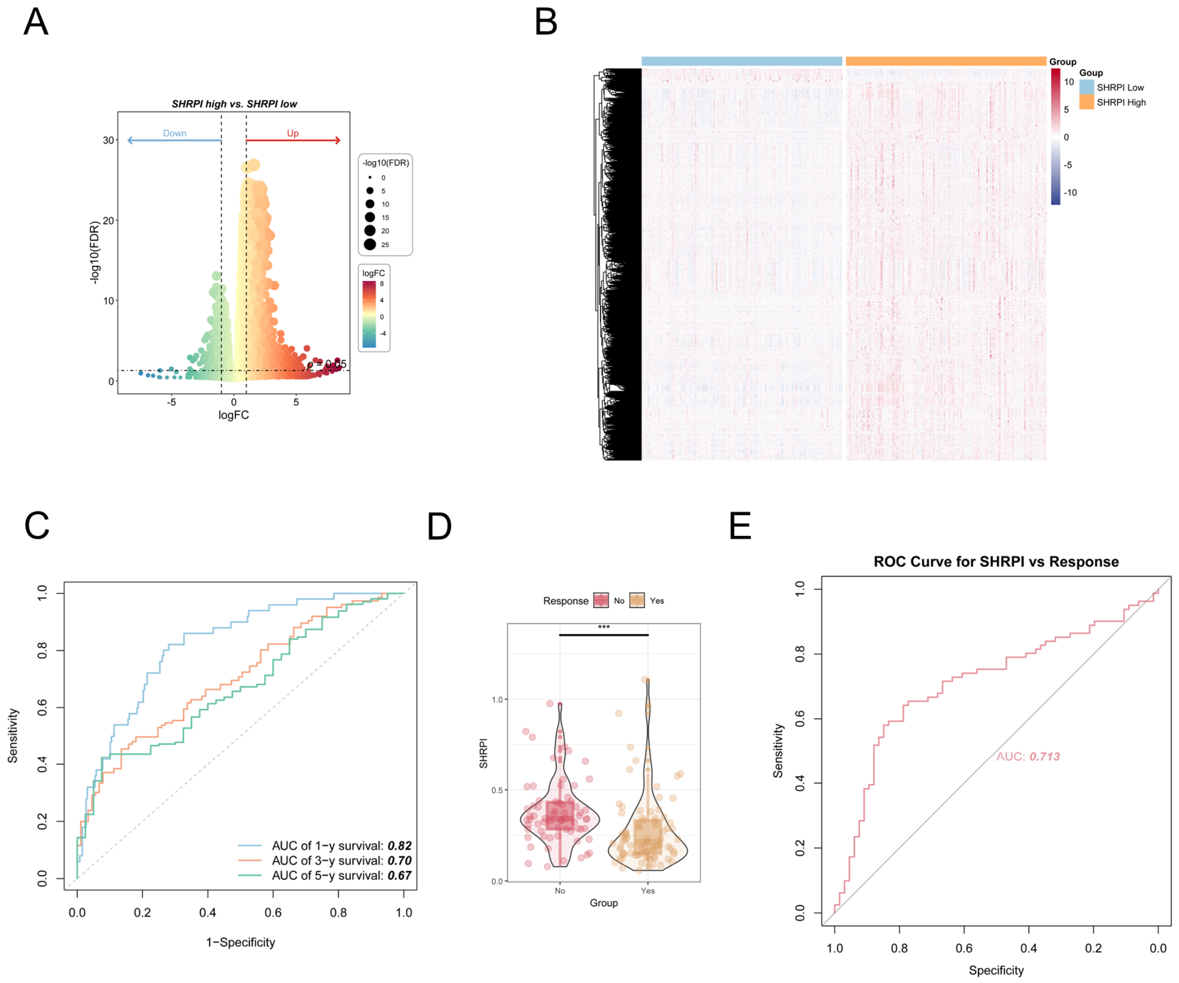

Supplementary Figure 4. Predictive performance of SHRPI for OS and TACE response. (**A, B**) Volcano plot (A) and heatmap (B) illustrating DEGs between low- and high-SHRPI groups. (**C**) Time-dependent ROC curves showing the SHRPI's predictive accuracy for 1-, 3-, and 5-year OS in the TCGA-LIHC cohort. (**D**) Comparison of SHRPI between TACE response and non-response groups. (**E**) ROC curve illustrating SHRPI's predictive performance for TACE response.

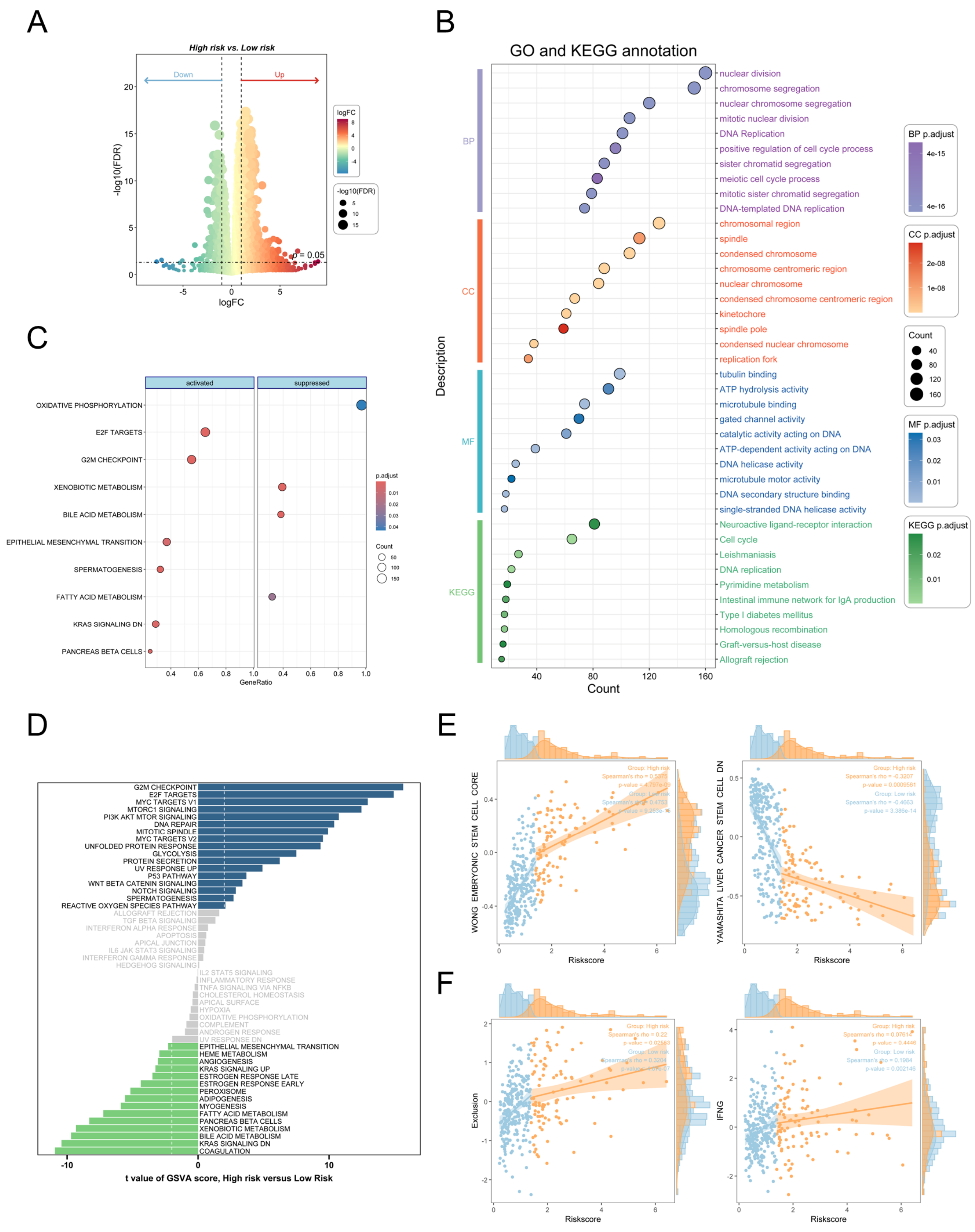

Supplementary Figure 5. Differential analysis, functional enrichment, and correlation with key gene signatures between low- and high-risk groups. (**A**) DEGs identified between low- and high-risk groups. (**B-D**) KEGG/GO (B), GSEA (C), and GSVA (D) enrichment analyses of the subgroups. (**E, F**) Correlation between SHRPI and Wong Embryonic Stem Cell Core, Yamashita Liver Cancer Stem

Cell Dn (E), and with key gene signatures (F), including Exclusion and IFNG (Spearman's correlation).

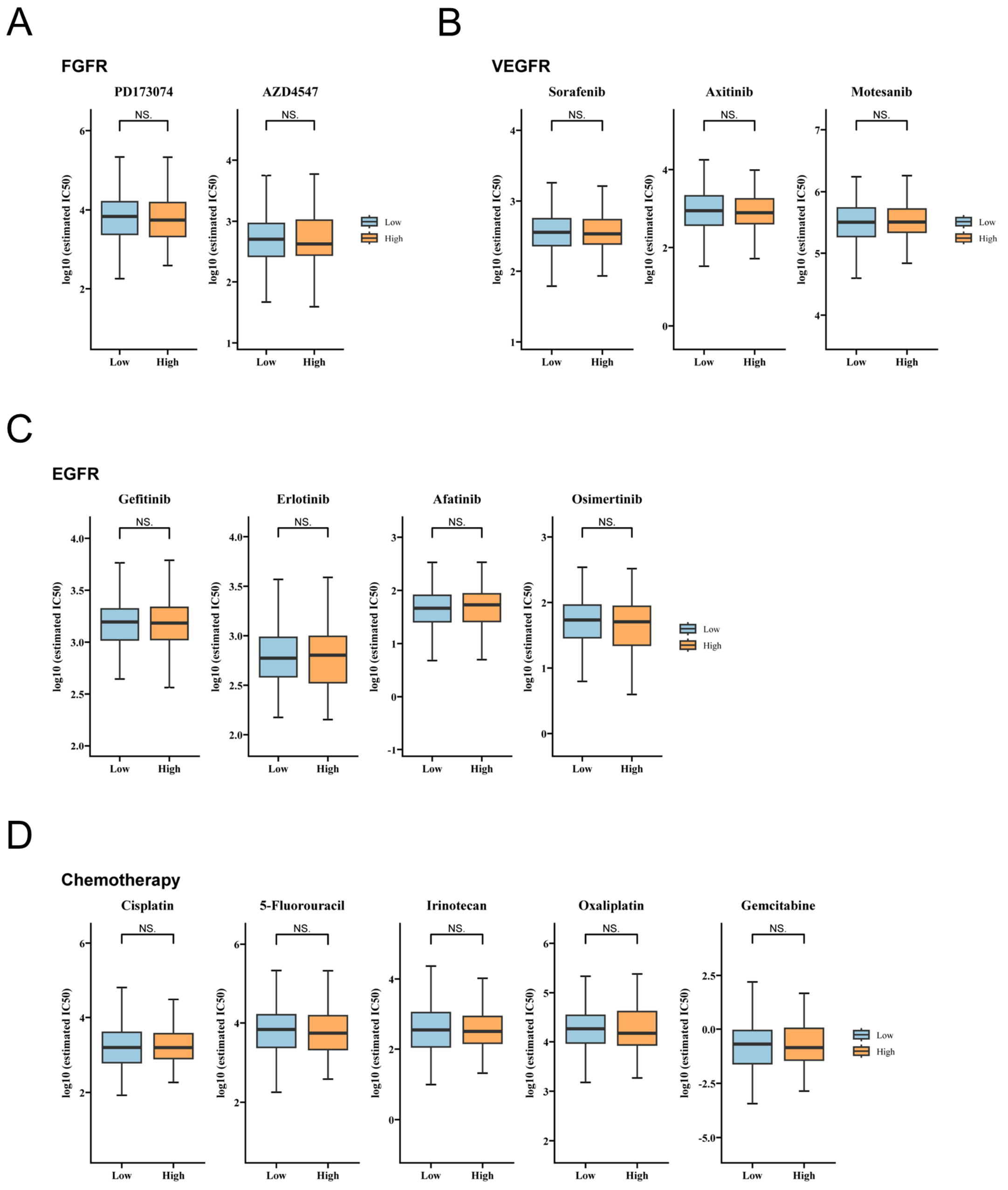

Supplementary Figure 6. Drug sensitivity comparisons between low- and high-risk groups. (**A-D**) Comparisons of estimated drug sensitivity between low- and high-risk groups for various drugs, including FGFR inhibitors (A), VEGFR inhibitors (B), EGFR inhibitors (C), and chemotherapy drugs (D).
