# Supplementary material for: Stemness- and hypoxia-based prognostic stratification index reveals G6PD as a regulator of hypoxia-driven stemness in hepatocellular carcinoma
Source: Front Immunol. 2025 Sep 19;16:1669275. doi: 10.3389/fimmu.2025.1669275 (PMC12491235; doi:10.3389/fimmu.2025.1669275)
Supplement: Supplementary file 2 [file DataSheet1.docx]

Supplementary Material

# Supplementary Data

Links to all figures and uncropped raw Western blot images for this manuscript:

**Figure 1 (**<https://www.jianguoyun.com/p/DVty2IoQtYLKDRig5YAGIAA>**)**

**Figure 2 (**<https://www.jianguoyun.com/p/DYcRa1MQtYLKDRiA7IAGIAA>**)**

**Figure 3 (**<https://www.jianguoyun.com/p/DQTYtigQtYLKDRiE7IAGIAA>**)**

**Figure 4 (**<https://www.jianguoyun.com/p/DUV8nPAQtYLKDRiG7IAGIAA>**)**

**Figure 5 (**<https://www.jianguoyun.com/p/DUKbuoEQtYLKDRiH7IAGIAA>**)**

**Figure 6 (**<https://www.jianguoyun.com/p/DUOF9bMQtYLKDRiJ7IAGIAA>**)**

**Figure 7 (**<https://www.jianguoyun.com/p/DUSBZJwQtYLKDRiK7IAGIAA>**)**

**Figure 8 (**<https://www.jianguoyun.com/p/Ddvzw3MQtYLKDRiM7IAGIAA>**)**

**Supplementary Figure 1 (**<https://www.jianguoyun.com/p/DcSwtvgQtYLKDRiO7IAGIAA>**)**

**Supplementary Figure 2 (**<https://www.jianguoyun.com/p/DXUnO8IQtYLKDRiP7IAGIAA>**)**

**Supplementary Figure 3 (**<https://www.jianguoyun.com/p/DTgmxmAQtYLKDRiQ7IAGIAA>**)**

**Supplementary Figure 4 (**<https://www.jianguoyun.com/p/Df9PsxMQtYLKDRiT7IAGIAA>**)**

**Supplementary Figure 5 (**<https://www.jianguoyun.com/p/Df0ZcjkQtYLKDRiU7IAGIAA>**)**

**Supplementary Figure 6 (**<https://www.jianguoyun.com/p/Df0ZcjkQtYLKDRiU7IAGIAA>**)**

**Western blot images (**<https://www.jianguoyun.com/p/DVoJQ7wQtYLKDRiX7IAGIAA>**)**
