# Supplementary material for: Stemness- and hypoxia-based prognostic stratification index reveals G6PD as a regulator of hypoxia-driven stemness in hepatocellular carcinoma
Source: Front Immunol. 2025 Sep 19;16:1669275. doi: 10.3389/fimmu.2025.1669275 (PMC12491235; doi:10.3389/fimmu.2025.1669275)
Supplement: Supplementary file 3 [file DataSheet2.docx]

Supplementary Material

# Supplementary Methods

## Lentiviral vector construction and infection

Plasmids were purchased from HZREPOBIO. Lentiviral plasmids encoding shRNAs targeting G6PD and a G6PD overexpression construct were used for viral packaging. Lentiviral particles were employed to infect the cells, and stable cell lines with successful gene expression were selected by treatment with 5 μg/mL puromycin (Selleck). All plasmid sequences are provided in Supplementary Table 3.

## Western blot analysis

Cells were collected and lysed using RIPA Lysis Buffer to obtain protein extracts. The protein concentration was determined using the BCA Protein Assay Kit (Thermo Fisher). Equal amounts of protein were separated by SDS-PAGE and transferred to a polyvinylidene fluoride (PVDF) membrane. The membrane was incubated overnight at 4°C with primary antibodies, followed by incubation with HRP-conjugated secondary antibodies at room temperature for 1 hour. Protein bands were detected using chemiluminescent reagents.

## Co-immunoprecipitation (Co-IP)

Cells (1×10^7^) were lysed in IP Lysis Buffer (Thermo Fisher) containing protease and phosphatase inhibitors. After centrifugation, the supernatant was collected, and a 3% aliquot was retained as the input control. Immunoprecipitation was performed by incubating the lysates with 1.5 μg of primary antibody and 20 μL of Protein A/G agarose beads at 4°C for 2-4 hours with gentle rotation. The antigen-antibody complexes bound to the beads were pelleted by centrifugation and eluted using Elution Buffer (Thermo Fisher) according to the manufacturer’s instructions. Input samples and immunoprecipitates were analyzed by Western blot.

All antibodies were used according to the manufacturer’s recommended protocols. For dilution, TBST buffer containing 5% bovine serum albumin was used, as detailed in Supplementary Table 4.

## Quantitative PCR (qPCR) analysis

Total RNA was extracted using the RNeasy Mini Kit (QIAGEN). The cDNA was generated using the Hiscript ® II RT SuperMix for qPCR (+gDNA wiper) kit and then amplified with ChamQ SYBR Color qPCR Master Mix (Vazyme). Real-time Quantitative PCR Detection was performed using the ABI 7500 Real-Time PCR System (Applied Biosystems). The sequences of primers are provided in Supplementary Table 5. The quantity of target cDNA was assessed by the converting the threshold cycle (CT) and normalizing it to β-actin. Each ample had three replicates.
