# Supplementary material for: Stemness- and hypoxia-based prognostic stratification index reveals G6PD as a regulator of hypoxia-driven stemness in hepatocellular carcinoma
Source: Front Immunol. 2025 Sep 19;16:1669275. doi: 10.3389/fimmu.2025.1669275 (PMC12491235; doi:10.3389/fimmu.2025.1669275)
Supplement: Supplementary Table 1 — Stemness- and hypoxia-related genes. [file Table1.docx]

Supplementary Material

# Supplementary Tables

**Supplementary Table 1.** Stemness- and hypoxia-related genes.

| **Gene** | **log2(FC)** | ***P* value** | **FDR** |
| --- | --- | --- | --- |
| STMN1 | 1.016417 | 2.76E-08 | 8.99E-08 |
| TOP2A | 1.017255 | 4.59E-06 | 6.61E-06 |
| RRM2 | 1.011769 | 2.24E-08 | 8.00E-08 |
| DUSP9 | 1.410215 | 9.74E-04 | 1.14E-03 |
| SPAG5 | 1.098786 | 3.67E-10 | 5.51E-09 |
| BIRC5 | 1.104047 | 2.95E-07 | 5.68E-07 |
| MYBL2 | 1.482726 | 8.23E-09 | 3.80E-08 |
| E2F1 | 1.22165 | 4.04E-08 | 1.26E-07 |
| CDC20 | 1.466458 | 2.24E-08 | 8.00E-08 |
| IGF2BP1 | 1.130549 | 2.09E-04 | 2.53E-04 |
| PTTG1 | 1.297705 | 1.07E-12 | 8.04E-11 |
| UBE2C | 1.151916 | 2.82E-06 | 4.14E-06 |
| UBE2S | 1.056948 | 2.39E-08 | 8.14E-08 |
| CDC6 | 1.087307 | 1.05E-07 | 2.56E-07 |
| CDCA5 | 1.099546 | 1.18E-09 | 1.48E-08 |
| CDT1 | 1.013262 | 5.29E-09 | 2.83E-08 |
| CCNA2 | 1.121732 | 1.73E-03 | 1.99E-03 |
| KIF4A | 1.135244 | 7.40E-07 | 1.29E-06 |
| SSX1 | 1.804772 | 2.69E-05 | 3.60E-05 |
| KIF2C | 1.283743 | 1.06E-07 | 2.56E-07 |
| PLK1 | 1.035478 | 2.03E-06 | 3.05E-06 |
| CDCA8 | 1.027001 | 1.02E-06 | 1.66E-06 |
| NCAPG | 1.131397 | 6.85E-08 | 1.98E-07 |
| HMMR | 1.020296 | 8.61E-09 | 3.80E-08 |
| NDC80 | 1.155598 | 3.39E-09 | 2.31E-08 |
| TROAP | 1.047902 | 1.47E-07 | 3.14E-07 |
| CDCA3 | 1.015799 | 9.91E-08 | 2.56E-07 |
| RMI2 | 1.079056 | 4.63E-07 | 8.27E-07 |
| NUF2 | 1.167943 | 1.40E-07 | 3.12E-07 |
| CENPM | 1.128401 | 4.47E-09 | 2.58E-08 |
| CENPW | 1.015687 | 1.41E-07 | 3.12E-07 |
| TRIP13 | 1.0538 | 8.70E-05 | 1.12E-04 |
| TTK | 1.271598 | 3.93E-09 | 2.46E-08 |
| SKA1 | 1.042145 | 9.04E-07 | 1.54E-06 |
| DEPDC1 | 1.31106 | 1.00E-07 | 2.56E-07 |
| EXO1 | 1.090042 | 6.22E-08 | 1.87E-07 |
| TEDC2 | 1.287522 | 3.91E-12 | 9.77E-11 |
| **Gene** | **log2(FC)** | ***P* value** | **FDR** |
| CDC25C | 1.298263 | 3.66E-12 | 9.77E-11 |
| SKA3 | 1.100478 | 1.57E-09 | 1.52E-08 |
| CDC25A | 1.255881 | 8.29E-09 | 3.80E-08 |
| ORC1 | 1.06878 | 9.84E-07 | 1.64E-06 |
| MCM10 | 1.09652 | 1.37E-06 | 2.10E-06 |
| IGF2BP3 | 1.094415 | 2.96E-03 | 3.23E-03 |
| KIF15 | 1.122363 | 2.92E-07 | 5.68E-07 |
| TUBA3C | 2.885923 | 2.95E-03 | 3.23E-03 |
| TRAIP | 1.080437 | 2.42E-09 | 1.81E-08 |
| PCLAF | 1.009633 | 1.98E-07 | 4.13E-07 |
| MAGEB2 | 1.824058 | 7.39E-03 | 7.70E-03 |
| CENPA | 1.014304 | 8.60E-06 | 1.22E-05 |
| LIN28B | 2.194416 | 8.84E-05 | 1.12E-04 |
| E2F2 | 1.017824 | 1.25E-07 | 2.93E-07 |
| SGO1 | 1.16337 | 1.62E-09 | 1.52E-08 |
| NEIL3 | 1.385809 | 9.52E-09 | 3.97E-08 |
| ZYG11A | 1.019836 | 2.19E-07 | 4.44E-07 |
| RAD54L | 1.055049 | 1.09E-05 | 1.51E-05 |
| EME1 | 1.160181 | 1.67E-08 | 6.59E-08 |
| FBXO43 | 1.028022 | 2.06E-09 | 1.72E-08 |
| SMC1B | 1.731961 | 2.54E-04 | 3.02E-04 |
| NAA11 | 1.956454 | 7.69E-03 | 7.90E-03 |
| TBX18 | 1.934438 | 1.34E-02 | 1.36E-02 |
| AUNIP | 1.087872 | 3.34E-07 | 6.11E-07 |
| RDM1 | 1.250585 | 2.21E-10 | 4.15E-09 |
| C12orf56 | 1.228406 | 3.06E-03 | 3.28E-03 |
| HPDL | 1.063325 | 1.40E-02 | 1.40E-02 |
| FAM72A | 1.030196 | 1.28E-06 | 2.00E-06 |
| FAM72B | 1.08671 | 1.18E-06 | 1.88E-06 |
| SOHLH2 | 2.314409 | 2.98E-03 | 3.23E-03 |
| DMC1 | 1.400964 | 4.51E-05 | 5.93E-05 |
| FAM72D | 1.173537 | 7.46E-08 | 2.07E-07 |
| CARD18 | 1.42506 | 9.08E-05 | 1.14E-04 |
| ELOVL3 | 2.056003 | 1.81E-04 | 2.23E-04 |
| FAM72C | 1.313218 | 3.25E-07 | 6.10E-07 |
| GNGT1 | 1.848006 | 1.33E-05 | 1.81E-05 |
| RNF186 | 1.433646 | 2.68E-03 | 3.04E-03 |
| KRTAP20-4 | 3.054391 | 6.96E-03 | 7.35E-03 |

**Supplementary Table 2.** Cox regression results of DEGs after collinearity filtering for SHRPI construction.

| **Gene** | **Coef** | **HR** | **HR_lower_95** | **HR_upper_95** | **Wald**  ***P* value** | **Likelihood**  ***P* value** | **Logrank**  ***P* value** |
| --- | --- | --- | --- | --- | --- | --- | --- |
| AFP | 1.79E-06 | 1.000002 | 1.000001 | 1.000003 | 4.27E-03 | 1.49E-02 | 3.41E-03 |
| STMN1 | 7.94E-05 | 1.000079 | 1.000050 | 1.000109 | 1.21E-07 | 7.68E-06 | 5.31E-08 |
| G6PD | 8.07E-05 | 1.000081 | 1.000058 | 1.000104 | 8.05E-12 | 1.10E-07 | 2.57E-14 |
| RRM2 | 1.93E-04 | 1.000193 | 1.000126 | 1.000260 | 1.35E-08 | 5.42E-06 | 2.37E-08 |
| DUSP9 | 7.24E-05 | 1.000072 | 1.000037 | 1.000108 | 5.29E-05 | 6.83E-04 | 2.78E-05 |
| BIRC5 | 1.53E-04 | 1.000153 | 1.000094 | 1.000211 | 3.15E-07 | 7.11E-05 | 2.11E-07 |
| NT5DC2 | 1.21E-04 | 1.000121 | 1.000070 | 1.000172 | 3.12E-06 | 1.96E-04 | 5.73E-07 |
| MYBL2 | 1.89E-04 | 1.000189 | 1.000123 | 1.000255 | 1.79E-08 | 2.65E-06 | 5.82E-09 |
| ASPM | 1.59E-04 | 1.000159 | 1.000075 | 1.000242 | 1.96E-04 | 1.71E-03 | 1.50E-04 |
| E2F1 | 1.42E-04 | 1.000142 | 1.000074 | 1.000209 | 3.83E-05 | 1.18E-03 | 2.98E-05 |
| TCF19 | 2.22E-04 | 1.000222 | 1.000097 | 1.000347 | 4.79E-04 | 1.41E-03 | 3.82E-04 |
| UBE2S | 3.59E-04 | 1.000359 | 1.000214 | 1.000503 | 1.15E-06 | 3.80E-05 | 7.48E-07 |
| FCGBP | 6.70E-05 | 1.000067 | 1.000015 | 1.000119 | 1.15E-02 | 3.13E-02 | 9.04E-03 |
| NPTX2 | 8.47E-05 | 1.000085 | 1.000042 | 1.000127 | 8.65E-05 | 1.38E-03 | 2.99E-05 |
| CCNA2 | 1.07E-04 | 1.000108 | 1.000022 | 1.000193 | 1.35E-02 | 3.35E-02 | 1.02E-02 |
| SPC24 | 3.23E-04 | 1.000323 | 1.000069 | 1.000577 | 1.28E-02 | 2.09E-02 | 1.25E-02 |
| DCAF4L2 | 1.92E-04 | 1.000192 | 1.000072 | 1.000313 | 1.75E-03 | 4.36E-03 | 1.47E-03 |
| SLC1A7 | 1.30E-04 | 1.000130 | 1.000080 | 1.000179 | 2.53E-07 | 1.35E-04 | 2.22E-08 |
| PRAME | 1.34E-04 | 1.000134 | 1.000051 | 1.000218 | 1.61E-03 | 6.83E-03 | 1.23E-03 |
| SSX1 | 1.37E-04 | 1.000137 | 1.000027 | 1.000247 | 1.46E-02 | 2.50E-02 | 1.36E-02 |
| CENPU | 3.20E-04 | 1.000320 | 1.000086 | 1.000554 | 7.31E-03 | 1.59E-02 | 6.99E-03 |
| ASF1B | 6.12E-04 | 1.000612 | 1.000360 | 1.000864 | 1.91E-06 | 2.57E-05 | 1.23E-06 |
| MEP1A | 1.46E-04 | 1.000146 | 1.000071 | 1.000221 | 1.37E-04 | 1.89E-03 | 4.70E-05 |
| CDKN3 | 5.89E-04 | 1.000590 | 1.000311 | 1.000868 | 3.35E-05 | 2.09E-04 | 3.01E-05 |
| AURKB | 3.16E-04 | 1.000316 | 1.000193 | 1.000440 | 5.28E-07 | 2.49E-04 | 6.88E-08 |
| HMMR | 1.01E-03 | 1.001011 | 1.000700 | 1.001322 | 1.92E-10 | 1.77E-08 | 6.06E-11 |
| PLBD1 | 2.20E-04 | 1.000220 | 1.000106 | 1.000334 | 1.53E-04 | 3.39E-03 | 5.52E-05 |
| HELLS | 5.57E-04 | 1.000557 | 1.000280 | 1.000834 | 8.00E-05 | 5.26E-04 | 6.22E-05 |
| TRIM71 | 2.25E-04 | 1.000225 | 1.000050 | 1.000399 | 1.16E-02 | 2.18E-02 | 1.06E-02 |
| RMI2 | 4.30E-04 | 1.000430 | 1.000168 | 1.000693 | 1.30E-03 | 4.82E-03 | 1.13E-03 |
| WDR62 | 5.46E-04 | 1.000546 | 1.000230 | 1.000863 | 7.18E-04 | 2.74E-03 | 6.61E-04 |
| NAALADL1 | 1.26E-04 | 1.000126 | 1.000048 | 1.000203 | 1.47E-03 | 1.40E-02 | 5.93E-04 |
| CENPM | 6.63E-04 | 1.000663 | 1.000351 | 1.000974 | 3.03E-05 | 1.96E-04 | 2.04E-05 |
| UHRF1 | 5.34E-04 | 1.000534 | 1.000295 | 1.000774 | 1.19E-05 | 2.30E-04 | 8.04E-06 |
| GTSF1 | 1.66E-04 | 1.000166 | 1.000009 | 1.000323 | 3.84E-02 | 5.68E-02 | 3.66E-02 |
| PNMA3 | 3.01E-04 | 1.000301 | 1.000156 | 1.000445 | 4.41E-05 | 5.81E-04 | 1.66E-05 |
| DEPDC1B | 3.24E-04 | 1.000324 | 1.000104 | 1.000545 | 3.96E-03 | 1.45E-02 | 3.14E-03 |
| **Gene** | **Coef** | **HR** | **HR_lower_95** | **HR_upper_95** | **Wald**  ***P* value** | **Likelihood**  ***P* value** | **Logrank**  ***P* value** |
| CTAG2 | 2.71E-04 | 1.000271 | 1.000103 | 1.000440 | 1.63E-03 | 6.24E-03 | 1.29E-03 |
| MAGEC1 | 2.08E-04 | 1.000208 | 1.000029 | 1.000386 | 2.27E-02 | 4.67E-02 | 2.06E-02 |
| TEDC2 | 1.12E-03 | 1.001122 | 1.000633 | 1.001610 | 6.73E-06 | 5.96E-05 | 5.00E-06 |
| CDCA7 | 4.08E-04 | 1.000408 | 1.000179 | 1.000636 | 4.69E-04 | 3.38E-03 | 2.52E-04 |
| CSAG1 | 4.58E-04 | 1.000458 | 1.000241 | 1.000676 | 3.54E-05 | 3.91E-04 | 2.34E-05 |
| PAGE2 | 1.64E-04 | 1.000165 | 1.000052 | 1.000277 | 4.31E-03 | 1.74E-02 | 2.94E-03 |
| CDC25C | 1.04E-03 | 1.001036 | 1.000544 | 1.001528 | 3.65E-05 | 2.26E-04 | 2.83E-05 |
| SHCBP1 | 8.10E-04 | 1.000810 | 1.000556 | 1.001064 | 4.04E-10 | 3.75E-06 | 4.63E-11 |
| CEP55 | 6.61E-04 | 1.000661 | 1.000432 | 1.000891 | 1.61E-08 | 2.36E-05 | 3.66E-10 |
| CACNG4 | 1.53E-04 | 1.000153 | 1.000006 | 1.000300 | 4.10E-02 | 9.84E-02 | 3.40E-02 |
| CDC25A | 1.25E-03 | 1.001254 | 1.000803 | 1.001706 | 5.01E-08 | 6.06E-06 | 2.69E-08 |
| SALL2 | 5.02E-04 | 1.000502 | 1.000088 | 1.000917 | 1.75E-02 | 2.81E-02 | 1.67E-02 |
| IGF2BP3 | 6.43E-04 | 1.000643 | 1.000370 | 1.000917 | 3.88E-06 | 8.44E-05 | 1.68E-06 |
| TUBA3C | 2.31E-04 | 1.000231 | 1.000111 | 1.000352 | 1.67E-04 | 2.96E-03 | 6.49E-05 |
| SALL4 | 5.42E-04 | 1.000542 | 1.000274 | 1.000810 | 7.33E-05 | 1.13E-03 | 3.56E-05 |
| TYMS | 1.32E-03 | 1.00132 | 1.000686 | 1.001954 | 4.44E-05 | 2.51E-04 | 3.48E-05 |
| PCLAF | 1.14E-03 | 1.001142 | 1.000628 | 1.001656 | 1.34E-05 | 2.22E-04 | 1.05E-05 |
| FAM133A | 4.28E-04 | 1.000428 | 1.000174 | 1.000683 | 9.85E-04 | 4.59E-03 | 7.21E-04 |
| MYO18B | 5.25E-04 | 1.000526 | 1.000251 | 1.000801 | 1.78E-04 | 1.15E-03 | 1.21E-04 |
| MAP7D2 | 2.17E-04 | 1.000217 | 1.000009 | 1.000425 | 4.05E-02 | 8.81E-02 | 3.53E-02 |
| HMGA2 | 2.34E-04 | 1.000234 | 1.00009 | 1.000378 | 1.45E-03 | 1.04E-02 | 6.63E-04 |
| ORC6 | 1.36E-03 | 1.001365 | 1.000879 | 1.001851 | 3.67E-08 | 7.49E-06 | 1.15E-08 |
| MMP1 | 8.49E-04 | 1.000849 | 1.000473 | 1.001225 | 9.57E-06 | 5.17E-04 | 8.05E-07 |
| SEMA3E | 2.68E-04 | 1.000268 | 1.000048 | 1.000488 | 1.69E-02 | 5.10E-02 | 1.52E-02 |
| LIN28B | 6.24E-04 | 1.000625 | 1.000223 | 1.001026 | 2.28E-03 | 8.16E-03 | 1.61E-03 |
| CSMD1 | 6.49E-04 | 1.000649 | 1.000243 | 1.001056 | 1.74E-03 | 7.10E-03 | 1.54E-03 |
| MCOLN3 | 4.82E-04 | 1.000482 | 1.000125 | 1.000839 | 8.11E-03 | 2.07E-02 | 7.06E-03 |
| GINS4 | 1.39E-03 | 1.001390 | 1.000586 | 1.002194 | 7.00E-04 | 3.44E-03 | 6.87E-04 |
| ESCO2 | 1.57E-03 | 1.001567 | 1.000986 | 1.002149 | 1.23E-07 | 7.53E-05 | 1.91E-07 |
| DQX1 | 5.70E-04 | 1.000570 | 1.000067 | 1.001074 | 2.63E-02 | 4.52E-02 | 2.51E-02 |
| SAPCD2 | 1.78E-03 | 1.001784 | 1.001221 | 1.002347 | 5.06E-10 | 1.85E-06 | 1.40E-10 |
| ZNF711 | 9.65E-04 | 1.000966 | 1.000314 | 1.001617 | 3.66E-03 | 8.51E-03 | 3.15E-03 |
| E2F7 | 1.31E-03 | 1.001308 | 1.000578 | 1.002039 | 4.46E-04 | 2.91E-03 | 4.68E-04 |
| NEIL3 | 2.76E-03 | 1.002767 | 1.001932 | 1.003602 | 7.96E-11 | 7.07E-08 | 5.18E-12 |
| PIMREG | 1.03E-03 | 1.001028 | 1.000472 | 1.001584 | 2.92E-04 | 3.72E-03 | 1.72E-04 |
| RTKN2 | 1.48E-03 | 1.001484 | 1.000832 | 1.002136 | 8.13E-06 | 5.90E-04 | 5.61E-06 |
| HAVCR1 | 3.95E-04 | 1.000395 | 1.000244 | 1.000546 | 2.92E-07 | 3.93E-04 | 1.46E-10 |
| FREM1 | 3.65E-04 | 1.000366 | 1.000051 | 1.000681 | 2.30E-02 | 6.54E-02 | 1.49E-02 |
| **Gene** | **Coef** | **HR** | **HR_lower_95** | **HR_upper_95** | **Wald**  ***P* value** | **Likelihood**  ***P* value** | **Logrank**  ***P* value** |
| TET1 | 2.31E-03 | 1.002309 | 1.001297 | 1.003323 | 7.69E-06 | 1.60E-04 | 4.25E-06 |
| ENPP6 | 5.20E-04 | 1.000520 | 1.000048 | 1.000992 | 3.07E-02 | 6.87E-02 | 2.72E-02 |
| UGT1A10 | 7.12E-04 | 1.000712 | 1.000387 | 1.001037 | 1.75E-05 | 8.80E-04 | 1.89E-06 |
| B3GALT2 | 5.45E-04 | 1.000545 | 1.000027 | 1.001063 | 3.93E-02 | 7.70E-02 | 3.30E-02 |
| TEX13C | 4.49E-04 | 1.000449 | 1.000233 | 1.000666 | 4.90E-05 | 3.98E-03 | 1.61E-06 |
| NPFFR2 | 8.64E-04 | 1.000865 | 1.000185 | 1.001545 | 1.27E-02 | 2.77E-02 | 1.10E-02 |
| FBXO43 | 4.92E-03 | 1.004928 | 1.002952 | 1.006907 | 9.84E-07 | 9.34E-06 | 6.12E-07 |
| CLIC3 | 1.14E-03 | 1.001139 | 1.000543 | 1.001736 | 1.79E-04 | 2.91E-03 | 6.96E-05 |
| RIBC2 | 2.93E-03 | 1.002939 | 1.001838 | 1.004042 | 1.65E-07 | 1.11E-05 | 4.49E-08 |
| RHEX | 4.30E-04 | 1.000431 | 1.000101 | 1.000761 | 1.05E-02 | 4.86E-02 | 5.66E-03 |
| SMC1B | 1.11E-03 | 1.001106 | 1.00042 | 1.001793 | 1.58E-03 | 9.70E-03 | 1.05E-03 |
| PKIA | 1.22E-03 | 1.001218 | 1.000491 | 1.001945 | 1.01E-03 | 7.44E-03 | 4.87E-04 |
| LYPD6 | 1.59E-03 | 1.001589 | 1.000643 | 1.002536 | 9.93E-04 | 4.59E-03 | 7.18E-04 |
| CLEC2L | 1.04E-03 | 1.001042 | 1.000469 | 1.001616 | 3.65E-04 | 4.76E-03 | 2.41E-04 |
| TFDP3 | 1.38E-03 | 1.001378 | 1.00063 | 1.002126 | 3.06E-04 | 1.61E-03 | 1.72E-04 |
| GABRA3 | 9.27E-04 | 1.000927 | 1.000496 | 1.001358 | 2.47E-05 | 2.30E-03 | 1.17E-06 |
| RFPL4B | 1.72E-03 | 1.001720 | 1.000843 | 1.002598 | 1.21E-04 | 1.12E-03 | 6.51E-05 |
| C5orf58 | 1.83E-03 | 1.001834 | 1.001092 | 1.002576 | 1.23E-06 | 1.69E-04 | 2.09E-07 |
| RCOR2 | 1.38E-03 | 1.001380 | 1.000797 | 1.001963 | 3.43E-06 | 5.65E-04 | 9.64E-08 |
| TRAM1L1 | 1.75E-03 | 1.001753 | 1.000528 | 1.002980 | 5.04E-03 | 1.29E-02 | 4.35E-03 |
| TMEM179 | 8.16E-04 | 1.000817 | 1.000236 | 1.001398 | 5.84E-03 | 2.23E-02 | 4.37E-03 |
| GDF10 | 1.60E-03 | 1.001604 | 1.000772 | 1.002437 | 1.57E-04 | 1.58E-03 | 6.92E-05 |
| NR0B1 | 7.92E-04 | 1.000792 | 1.000351 | 1.001234 | 4.38E-04 | 5.62E-03 | 1.44E-04 |
| RDM1 | 5.62E-03 | 1.005633 | 1.003154 | 1.008117 | 8.15E-06 | 9.04E-05 | 6.88E-06 |
| MPP2 | 3.98E-03 | 1.003984 | 1.002608 | 1.005361 | 1.31E-08 | 8.25E-06 | 1.45E-09 |
| CSAG3 | 2.64E-03 | 1.002646 | 1.001052 | 1.004242 | 1.13E-03 | 4.33E-03 | 9.05E-04 |
| MDGA2 | 2.38E-03 | 1.002384 | 1.000777 | 1.003994 | 3.63E-03 | 1.01E-02 | 3.35E-03 |
| CERS1 | 1.37E-03 | 1.001371 | 1.000096 | 1.002648 | 3.51E-02 | 5.74E-02 | 3.24E-02 |
| MYB | 3.43E-03 | 1.003434 | 1.001496 | 1.005376 | 5.09E-04 | 4.90E-03 | 2.65E-04 |
| CNTNAP4 | 3.21E-03 | 1.003215 | 1.000918 | 1.005517 | 6.05E-03 | 1.14E-02 | 5.46E-03 |
| C12orf56 | 2.64E-03 | 1.002648 | 1.000926 | 1.004372 | 2.57E-03 | 6.75E-03 | 2.04E-03 |
| DCAF8L1 | 1.42E-03 | 1.001418 | 1.000907 | 1.001929 | 5.17E-08 | 2.42E-04 | 1.99E-12 |
| ELOVL4 | 1.72E-03 | 1.001719 | 1.000171 | 1.003268 | 2.95E-02 | 6.57E-02 | 2.51E-02 |
| SEPTIN14 | 1.07E-03 | 1.001068 | 1.000329 | 1.001808 | 4.62E-03 | 2.36E-02 | 1.88E-03 |
| CDH9 | 1.90E-03 | 1.001901 | 1.000504 | 1.003300 | 7.63E-03 | 2.25E-02 | 5.97E-03 |
| GPR19 | 6.73E-03 | 1.006753 | 1.00264 | 1.010882 | 1.27E-03 | 3.19E-03 | 1.07E-03 |
| MAGEB1 | 1.52E-03 | 1.001523 | 1.000248 | 1.002800 | 1.92E-02 | 4.36E-02 | 1.56E-02 |
| SOHLH2 | 1.69E-03 | 1.001690 | 1.000605 | 1.002776 | 2.25E-03 | 1.20E-02 | 1.15E-03 |
| CRISP2 | 5.77E-03 | 1.005783 | 1.002957 | 1.008617 | 5.90E-05 | 1.71E-03 | 7.27E-06 |
| **Gene** | **Coef** | **HR** | **HR_lower_95** | **HR_upper_95** | **Wald**  ***P* value** | **Likelihood**  ***P* value** | **Logrank**  ***P* value** |
| GLB1L3 | 2.05E-03 | 1.002056 | 1.000344 | 1.003771 | 1.85E-02 | 4.57E-02 | 1.56E-02 |
| FABP6 | 1.65E-03 | 1.001652 | 1.00077 | 1.002534 | 2.40E-04 | 4.32E-03 | 5.96E-05 |
| TEX15 | 2.38E-03 | 1.002387 | 1.001531 | 1.003243 | 4.42E-08 | 6.74E-05 | 7.71E-11 |
| C11orf53 | 1.71E-03 | 1.001714 | 1.000949 | 1.002480 | 1.13E-05 | 1.63E-03 | 1.17E-06 |
| DLX6 | 2.91E-03 | 1.002912 | 1.00075 | 1.005079 | 8.26E-03 | 2.12E-02 | 6.87E-03 |
| FAM72D | 1.31E-02 | 1.013167 | 1.008927 | 1.017425 | 9.75E-10 | 7.11E-07 | 2.37E-10 |
| CARD18 | 3.84E-03 | 1.003849 | 1.000966 | 1.006741 | 8.85E-03 | 2.23E-02 | 7.75E-03 |
| STEAP1B | 1.79E-03 | 1.001789 | 1.000294 | 1.003287 | 1.90E-02 | 4.05E-02 | 1.52E-02 |
| KISS1R | 2.65E-03 | 1.002653 | 1.001022 | 1.004286 | 1.42E-03 | 8.93E-03 | 7.66E-04 |
| SSTR3 | 2.01E-03 | 1.002017 | 1.000007 | 1.004032 | 4.93E-02 | 8.58E-02 | 4.38E-02 |
| KCNC1 | 1.85E-03 | 1.001849 | 1.000447 | 1.003253 | 9.75E-03 | 3.04E-02 | 7.00E-03 |
| ELOVL3 | 2.52E-03 | 1.002523 | 1.000598 | 1.004451 | 1.02E-02 | 3.47E-02 | 7.04E-03 |
| ART5 | 3.40E-03 | 1.003406 | 1.001164 | 1.005654 | 2.90E-03 | 1.49E-02 | 1.88E-03 |
| GAGE2A | 2.40E-03 | 1.002399 | 1.000745 | 1.004056 | 4.46E-03 | 1.96E-02 | 2.38E-03 |
| FAM183A | 3.13E-03 | 1.003139 | 1.00102 | 1.005262 | 3.67E-03 | 2.34E-02 | 1.76E-03 |
| ISL2 | 4.60E-03 | 1.004607 | 1.001068 | 1.008160 | 1.07E-02 | 2.29E-02 | 9.06E-03 |
| AC139491.7 | 4.49E-03 | 1.004503 | 1.001108 | 1.007908 | 9.29E-03 | 2.95E-02 | 7.70E-03 |
| LBHD2 | 1.76E-03 | 1.001761 | 1.000035 | 1.003490 | 4.55E-02 | 9.39E-02 | 4.02E-02 |
| FAM72C | 1.21E-02 | 1.012158 | 1.007568 | 1.016769 | 1.88E-07 | 8.07E-05 | 5.16E-08 |
| OR56A3 | 2.32E-03 | 1.002320 | 1.000931 | 1.003712 | 1.06E-03 | 1.80E-02 | 1.76E-04 |
| POU3F2 | 2.19E-03 | 1.002193 | 1.000626 | 1.003763 | 6.07E-03 | 2.86E-02 | 2.80E-03 |
| GNGT1 | 1.05E-02 | 1.010548 | 1.006328 | 1.014786 | 8.91E-07 | 6.64E-05 | 3.53E-07 |
| NKX3_2 | 2.92E-03 | 1.002924 | 1.000657 | 1.005197 | 1.15E-02 | 4.09E-02 | 8.04E-03 |
| CT55 | 4.75E-03 | 1.004757 | 1.001465 | 1.008061 | 4.60E-03 | 2.41E-02 | 2.72E-03 |
| CT83 | 4.54E-03 | 1.004553 | 1.001204 | 1.007913 | 7.68E-03 | 2.57E-02 | 5.77E-03 |
| ALX1 | 6.98E-03 | 1.007004 | 1.004351 | 1.009664 | 2.16E-07 | 1.73E-04 | 9.96E-09 |
| ZNF730 | 8.66E-03 | 1.008694 | 1.002535 | 1.014891 | 5.60E-03 | 1.74E-02 | 4.90E-03 |
| AKAIN1 | -2.07E-02 | 0.979534 | 0.96116 | 0.998260 | 3.23E-02 | 2.22E-03 | 2.39E-02 |
| SLITRK1 | 3.16E-03 | 1.003170 | 1.000295 | 1.006052 | 3.06E-02 | 6.54E-02 | 2.46E-02 |
| ZNF723 | 4.70E-03 | 1.004710 | 1.000104 | 1.009338 | 4.50E-02 | 7.98E-02 | 4.19E-02 |
| SPAG6 | 5.37E-03 | 1.005385 | 1.001131 | 1.009657 | 1.30E-02 | 3.59E-02 | 9.44E-03 |
| DDN | 1.41E-02 | 1.014228 | 1.007901 | 1.020594 | 9.64E-06 | 9.84E-04 | 5.75E-07 |
| TLX2 | 9.12E-03 | 1.009165 | 1.004177 | 1.014177 | 3.07E-04 | 4.08E-03 | 1.12E-04 |
| AIRE | 5.08E-03 | 1.005093 | 1.000197 | 1.010013 | 4.15E-02 | 7.91E-02 | 3.50E-02 |
| LRRIQ1 | 7.61E-03 | 1.007637 | 1.001616 | 1.013694 | 1.28E-02 | 3.69E-02 | 1.05E-02 |
| ACRV1 | 3.27E-02 | 1.033286 | 1.015974 | 1.050894 | 1.46E-04 | 1.28E-03 | 1.37E-04 |
| SPATA31D1 | 4.49E-03 | 1.004500 | 1.00063 | 1.008384 | 2.26E-02 | 5.56E-02 | 1.77E-02 |
| VCX3B | 1.15E-02 | 1.011606 | 1.007444 | 1.015785 | 4.12E-08 | 1.19E-04 | 3.39E-11 |
| **Gene** | **Coef** | **HR** | **HR_lower_95** | **HR_upper_95** | **Wald**  ***P* value** | **Likelihood**  ***P* value** | **Logrank**  ***P* value** |
| TGIF2LX | 9.35E-03 | 1.009392 | 1.001485 | 1.017362 | 1.98E-02 | 4.05E-02 | 1.82E-02 |
| OR8G5 | 1.13E-02 | 1.011358 | 1.001163 | 1.021656 | 2.89E-02 | 5.12E-02 | 2.63E-02 |
| ARMC3 | 9.12E-03 | 1.009161 | 1.004591 | 1.013753 | 8.23E-05 | 4.23E-03 | 3.35E-06 |
| PLAC1 | 9.08E-03 | 1.009121 | 1.003367 | 1.014909 | 1.86E-03 | 1.99E-02 | 4.02E-04 |
| C19orf84 | 1.32E-02 | 1.013286 | 1.005004 | 1.021635 | 1.62E-03 | 1.20E-02 | 9.42E-04 |
| NXPH2 | 5.24E-03 | 1.005252 | 1.000184 | 1.010345 | 4.22E-02 | 9.42E-02 | 3.18E-02 |
| ANKRD7 | 2.42E-02 | 1.024534 | 1.010997 | 1.038252 | 3.55E-04 | 2.96E-03 | 1.69E-04 |
| TCF24 | 1.11E-02 | 1.011118 | 1.001218 | 1.021115 | 2.76E-02 | 7.30E-02 | 2.11E-02 |
| GFY | 1.11E-02 | 1.011165 | 1.001218 | 1.021210 | 2.77E-02 | 7.58E-02 | 2.03E-02 |
| OR8A1 | 3.48E-02 | 1.035400 | 1.018711 | 1.052363 | 2.72E-05 | 3.33E-04 | 1.45E-05 |
| PGLYRP4 | 1.27E-02 | 1.012762 | 1.005019 | 1.020565 | 1.20E-03 | 9.43E-03 | 4.23E-04 |
| STXBP5L | 2.06E-02 | 1.020854 | 1.010947 | 1.030859 | 3.36E-05 | 2.38E-03 | 9.50E-06 |
| NKX2_8 | 2.76E-02 | 1.027986 | 1.005965 | 1.050490 | 1.25E-02 | 3.29E-02 | 1.06E-02 |
| ZNF280A | 2.64E-02 | 1.026733 | 1.013377 | 1.040265 | 7.85E-05 | 2.36E-03 | 1.69E-05 |
| KCNG3 | 2.46E-02 | 1.024866 | 1.006477 | 1.043592 | 7.84E-03 | 2.15E-02 | 6.66E-03 |
| RIPPLY2 | 2.95E-02 | 1.029986 | 1.014287 | 1.045927 | 1.63E-04 | 1.90E-03 | 6.56E-05 |
| KRTAP4_1 | 1.18E-02 | 1.011834 | 1.003611 | 1.020124 | 4.72E-03 | 2.21E-02 | 2.19E-03 |
| RNASEH2A | 5.57E-02 | 1.057293 | 1.017064 | 1.099114 | 4.88E-03 | 1.15E-02 | 4.38E-03 |
| GRID2 | 3.37E-02 | 1.034295 | 1.021004 | 1.047758 | 3.22E-07 | 1.25E-04 | 4.63E-09 |
| PANX3 | 1.70E-02 | 1.017193 | 1.002773 | 1.031821 | 1.93E-02 | 6.28E-02 | 1.20E-02 |
| AVPR1B | 2.25E-02 | 1.022744 | 1.006492 | 1.039258 | 5.93E-03 | 2.93E-02 | 4.58E-03 |
| KCNV1 | 1.38E-02 | 1.013909 | 1.001632 | 1.026336 | 2.63E-02 | 6.83E-02 | 2.04E-02 |
| MAGEB10 | 1.29E-02 | 1.012990 | 1.002063 | 1.024037 | 1.97E-02 | 5.94E-02 | 1.20E-02 |
| OR5H6 | 4.19E-02 | 1.042744 | 1.019844 | 1.066158 | 2.21E-04 | 2.90E-03 | 1.04E-04 |
| CST8 | 2.57E-02 | 1.026007 | 1.001578 | 1.051032 | 3.68E-02 | 7.24E-02 | 3.20E-02 |
| TP53TG3D | 2.85E-02 | 1.028877 | 1.008967 | 1.049179 | 4.30E-03 | 2.03E-02 | 2.92E-03 |
| XAGE1A | 2.72E-02 | 1.027597 | 1.010871 | 1.044600 | 1.15E-03 | 2.09E-02 | 2.77E-04 |
| KRT79 | 2.71E-02 | 1.027467 | 1.008183 | 1.047119 | 5.06E-03 | 2.66E-02 | 2.12E-03 |
| TEX13D | 3.31E-02 | 1.033659 | 1.010635 | 1.057209 | 3.97E-03 | 1.74E-02 | 2.49E-03 |
| SSX4B | 2.90E-02 | 1.029416 | 1.000004 | 1.059694 | 5.00E-02 | 9.53E-02 | 4.25E-02 |
| RBMY1E | 5.31E-02 | 1.054514 | 1.013202 | 1.097510 | 9.24E-03 | 2.65E-02 | 8.36E-03 |
| NPSR1 | 8.52E-02 | 1.088915 | 1.009453 | 1.174632 | 2.76E-02 | 4.94E-02 | 2.65E-02 |
| BOLL | 6.57E-02 | 1.067898 | 1.034285 | 1.102603 | 5.68E-05 | 2.80E-03 | 6.91E-06 |
| TGIF2LY | 3.89E-02 | 1.039709 | 1.011839 | 1.068347 | 4.97E-03 | 2.76E-02 | 3.38E-03 |
| MAGEA2 | 7.55E-02 | 1.078468 | 1.045112 | 1.112888 | 2.44E-06 | 6.76E-04 | 5.25E-07 |
| KRTAP19_1 | 7.28E-02 | 1.075565 | 1.036668 | 1.115922 | 1.06E-04 | 3.29E-03 | 2.62E-05 |
| MS4A13 | 3.64E-02 | 1.037081 | 1.000085 | 1.075445 | 4.95E-02 | 1.06E-01 | 4.23E-02 |
| MCHR2 | 6.16E-02 | 1.063520 | 1.023390 | 1.105223 | 1.70E-03 | 2.51E-02 | 7.31E-04 |
| CD200R1L | 1.20E-01 | 1.127689 | 1.029956 | 1.234696 | 9.37E-03 | 1.85E-02 | 8.30E-03 |
| **Gene** | **Coef** | **HR** | **HR_lower_95** | **HR_upper_95** | **Wald**  ***P* value** | **Likelihood**  ***P* value** | **Logrank**  ***P* value** |
| DEFB126 | 7.92E-02 | 1.082453 | 1.031100 | 1.136364 | 1.40E-03 | 9.92E-03 | 7.00E-04 |
| POTEC | 4.74E-02 | 1.048573 | 1.005710 | 1.093262 | 2.59E-02 | 6.42E-02 | 2.11E-02 |
| AADACL3 | 5.63E-02 | 1.057933 | 1.021090 | 1.096106 | 1.85E-03 | 1.30E-02 | 8.47E-04 |
| OR52E6 | 1.63E-01 | 1.176492 | 1.083021 | 1.278031 | 1.19E-04 | 1.09E-03 | 7.00E-05 |
| KRT25 | 8.07E-02 | 1.083992 | 1.003167 | 1.171329 | 4.14E-02 | 8.71E-02 | 3.41E-02 |
| MAGEA2B | 1.59E-01 | 1.171811 | 1.101753 | 1.246323 | 4.64E-07 | 1.10E-04 | 3.62E-08 |
| DMRTC2 | 8.33E-02 | 1.086888 | 1.013948 | 1.165074 | 1.87E-02 | 4.89E-02 | 1.49E-02 |
| TAS2R38 | 1.05E-01 | 1.111188 | 1.044529 | 1.182100 | 8.37E-04 | 8.66E-03 | 3.20E-04 |
| PDHA2 | 1.01E-01 | 1.106745 | 1.03896 | 1.178953 | 1.66E-03 | 1.52E-02 | 6.34E-04 |
| CTAG1A | 1.59E-01 | 1.172689 | 1.07207 | 1.282752 | 5.01E-04 | 4.48E-03 | 2.73E-04 |
| OR52E5 | 2.60E-01 | 1.297224 | 1.158275 | 1.452841 | 6.74E-06 | 2.85E-04 | 3.56E-06 |
| GALNTL5 | 2.17E-01 | 1.241779 | 1.143676 | 1.348297 | 2.51E-07 | 1.59E-04 | 1.81E-08 |
| ADAM7 | 2.46E-01 | 1.279402 | 1.029021 | 1.590707 | 2.66E-02 | 4.92E-02 | 2.34E-02 |
| TPTE | 1.96E-01 | 1.216209 | 1.056656 | 1.399853 | 6.37E-03 | 2.13E-02 | 5.62E-03 |
| MMD2 | 1.40E-01 | 1.150649 | 1.051415 | 1.25925 | 2.29E-03 | 1.28E-02 | 1.22E-03 |
| OR52E4 | 2.44E-01 | 1.27688 | 1.006924 | 1.619212 | 4.37E-02 | 6.33E-02 | 4.17E-02 |
| OR10D3 | 3.43E-01 | 1.40868 | 1.194721 | 1.660956 | 4.57E-05 | 1.00E-03 | 1.37E-05 |
| CSH2 | 2.35E-01 | 1.265115 | 1.087175 | 1.472179 | 2.36E-03 | 1.46E-02 | 1.28E-03 |
| TEX13B | 3.40E-01 | 1.404499 | 1.169955 | 1.686063 | 2.69E-04 | 2.65E-03 | 1.74E-04 |
| SPANXN3 | 2.93E-01 | 1.340156 | 1.166781 | 1.539294 | 3.44E-05 | 1.82E-03 | 9.19E-06 |
| KRTAP16_1 | 3.95E-01 | 1.484052 | 1.145632 | 1.922441 | 2.79E-03 | 1.02E-02 | 2.34E-03 |
| FAM47A | 5.53E-01 | 1.738482 | 1.454772 | 2.077521 | 1.17E-09 | 2.14E-05 | 8.88E-14 |
| OR52L1 | 4.34E-01 | 1.543588 | 1.204454 | 1.978211 | 6.04E-04 | 3.97E-03 | 3.29E-04 |
| OR56A5 | 3.03E-01 | 1.353796 | 1.005809 | 1.822179 | 4.57E-02 | 8.21E-02 | 4.06E-02 |
| SPEM1 | 4.57E-01 | 1.579706 | 1.055733 | 2.363733 | 2.62E-02 | 4.69E-02 | 2.39E-02 |
| OR2M3 | 5.86E-01 | 1.796465 | 1.133377 | 2.847494 | 1.27E-02 | 2.63E-02 | 1.08E-02 |
| USP29 | 6.22E-01 | 1.863065 | 1.240253 | 2.798632 | 2.73E-03 | 1.20E-02 | 1.82E-03 |
| OR9I1 | 5.20E-01 | 1.682828 | 1.093944 | 2.588716 | 1.79E-02 | 3.97E-02 | 1.49E-02 |
| TP53TG3 | 1.02E+00 | 2.775905 | 1.806098 | 4.266461 | 3.23E-06 | 1.76E-04 | 3.99E-07 |
| KRTAP9_1 | 8.69E-01 | 2.384783 | 1.049237 | 5.420306 | 3.80E-02 | 6.69E-02 | 3.23E-02 |

**Supplementary Table 3.** Detailed information of oligonucleotides used in experiments.

| Oligonucleotides | Company |
| --- | --- |
| **shRNA targeting sequence**  **G6PD 1#**  ACCGGTCCAAGATGATGACCAAGAAGCTTCAAGAGAGCTTCTTGGTCATCATCTTGGTTTTTTGAATTC  **G6PD 2#**  ACCGGTAGAAGGTCAAGGTGTTGAAATTTCAAGAGAATTTCAACACCTTGACCTTCTTTTTTTGAATTC  **G6PD 3#**  ACCGGTGCAAGGAGATGGTGCAGAACCTTCAAGAGAGGTTCTGCACCATCTCCTTGCTTTTTTGAATTC | HZREPOBIO |
| **pLV3-CMV-G6PD (human)-3×Myc-Puro** | HZREPOBIO |
| **pT3-EF1α-c-Met** | HZREPOBIO |
| **pT3-myr-AKT-HA** | HZREPOBIO |
| **pT3-EF1α-MYC (MCS)** | HZREPOBIO |
| **m-G6PD pT3-EF1α-MYC** | HZREPOBIO |
| **pCMV(CAT)T7-SB100** | HZREPOBIO |

**Supplementary Table 4.** Detailed information of antibodies used in experiments.

| Antibodies | Company | Product code | Dilution |
| --- | --- | --- | --- |
| β-actin antibody | Proteintech | Cat# 60008-1-Ig | Western Blot: 1/10000 |
| Goat anti-mouse IgG (H+L) | Proteintech | Cat# SA00001-1 | Western Blot: 1/10000 |
| Goat anti-rabbit IgG (H+L) | Proteintech | Cat# SA00001-2 | Western Blot: 1/10000 |
| Anti-HIF-1 alpha | Abcam | Car#ab179483 | Western Blot: 1/2000 |
| HIF-1α antibody | Proteintech | Cat# 20960-1-AP | Immunoprecipitation: 1.5-2 μg for 1.0 mg of total protein lysate |
| Rabbit IgG | Abcam | Cat# ab172730 | Immunoprecipitation: 1.5-2 μg for 1.0 mg of total protein lysate |
| Anti- Glucose 6 Phosphate Dehydrogenase | Abcam | Car# ab210702 | Western Blot: 1/1000  Immunoprecipitation: 1.5-2 μg for 1.0 mg of total protein lysate |

**Supplementary Table 5.** Detailed information of primers used in experiments.

| Gene | Forward primer (5’-3’) | Reverse primer (5’-3’) |
| --- | --- | --- |
| β-actin | CATCCACGAAACTACCTTCAACTCC | GAGCCGCCGATCCACACG |
| CD24 | TGAAGAACATGTGAGAGGTTTGAC | GAAAACTGAATCTCCATTCCACAA |
| CD44 | CTGCCGCTTTGCAGGTGTA | CATTGTGGGCAAGGTGCTATT |
| OCT3/4 | CTTGCTGCAGAAGTGGGTGGAGGAA | CTGCAGTGTGGGTTTCGGGCA |
